# Chronic Fatty Acid Depletion Induces Uncoupling Protein 1 (UCP1) Expression to Coordinate Mitochondrial Inducible Proton Leak in a Human-Brown-Adipocyte Model

**DOI:** 10.3390/cells11132038

**Published:** 2022-06-27

**Authors:** Yukimasa Takeda, Ping Dai

**Affiliations:** Department of Cellular Regenerative Medicine, Graduate School of Medical Science, Kyoto Prefectural University of Medicine, 465 Kajii-cho, Kawaramachi-Hirokoji, Kamigyo-ku, Kyoto 602-8566, Japan

**Keywords:** human brown adipocytes, uncoupling protein 1, transcriptome analysis, mitochondria, lipid metabolism, obesity

## Abstract

Thermogenic brown fat contributes to metabolic health in adult humans. Obese conditions are known to repress adipose-tissue browning and its activity. Herein, we found that chronic fatty acid (FA) depletion induced uncoupling protein 1 (UCP1) expression in the chemical-compound-induced brown adipocytes (ciBAs). The ciBAs, converted from human dermal fibroblasts under FA-free conditions, had low intracellular triglyceride levels and strongly activated UCP1 expression. Prolonged treatment with carnitine also reduced triglyceride accumulation and induced UCP1 expression. Transcriptome analysis revealed that the *UCP1* induction was accompanied by the activation of lipid metabolic genes. The FA-depleted conditions repressed mitochondrial proton-leak activity and mitochondrial membrane potential (MMP), despite maintaining a high UCP1 expression. The evidence suggested that UCP1 expression was induced to compensate for the proton-leak activity under low MMP. Our study reports a regulatory mechanism underlying UCP1 expression and mitochondrial-energy status in human brown adipocytes under different nutritional conditions.

## 1. Introduction

A disrupted balance between caloric intake and energy expenditure causes obesity and related metabolic diseases. White adipose tissue (WAT) stores excess energy as triglycerides, whereas brown adipose tissue (BAT) is specialized to consume glucose and fatty acids (FAs) as substrates for heat production [1]. Uncoupling protein 1 (UCP1) is specifically expressed in BAT and localized on mitochondrial inner membranes [2]. UCP1 dissipates the mitochondrial proton gradient by uncoupling cellular respiration and mitochondrial adenosine triphosphate (ATP) synthesis, which is responsible for the inducible proton leak and thermogenesis in BAT. In response to cold exposure, BAT activates UCP1 expression and FA oxidation to resist hypothermia in mammals, including rodents and newborn humans. The presence of brown fat in adult humans has been identified using 18F-fluorodeoxyglucose positron-emission-tomography and computed-tomography (18F-FDG PET/CT) scans [3,4,5]. Human BAT is sporadically located in cervical, supraclavicular, and paravertebral adipose depots. Human brown/beige adipocytes are either differentiated de novo from progenitor cells with a vascular smooth-muscle-gene signature or converted from dormant white-like adipocytes in response to external and pharmacological stimuli, referred to as adipocyte browning [6,7]. A retrospective large cohort study using 18F-FDG PET/CT scans revealed that brown fat is significantly associated with a lower risk of type 2 diabetes, dyslipidemia, coronary artery disease, and hypertension [8]. The observation was supported by improved levels of blood glucose, triglycerides, and high-density lipoproteins in patients with brown fat. Besides, several other studies also indicated that human BAT potentially had beneficial effects on metabolic health [9,10,11,12,13]. Therefore, the manipulation of BAT may provide a therapeutic intervention strategy to combat obesity and related metabolic diseases.

Accumulating evidence regarding the quantification of human BAT using 18F-FDG PET/CT scans has indicated that their levels are inversely correlated with the body mass index (BMI) in both cold and thermoneutral conditions [3,4,5,14,15,16,17]. The detailed and careful comparison between young healthy, obese, and lean subjects indicated that obese individuals had less metabolically activated BAT in six anatomically distinct BAT-containing fat depots [14]. In addition, the effects of cold exposure in BAT were negatively correlated with obesity and BMI [15,17,18]. Human brown adipocytes are known to resemble beige adipocytes generated within WAT in rodents [1,2]. Rodent beige adipocytes in obese WAT depots also reduced UCP1 expression [19,20]. Thus, lean and obese metabolic conditions similarly affected the amount and activity of both human and rodent beige adipocytes, suggesting that a common regulatory mechanism may be present. Several studies have reported that the less-active BAT in obese mice is caused by mediating multiple systemic factors, such as inflammatory cytokines (interleukin-25 and -27), growth factors (transforming growth factor-β), Notch signaling, selenoprotein P, and sympathetic nervous activity [2,21,22,23,24,25]. However, other metabolic factors regulating lean and obese brown adipocytes have not been fully identified.

The availability of human primary brown fat is limited, owing to its fragile nature and the invasiveness of biopsies [26]. Therefore, we have previously developed a technique to prepare chemical-compound-induced brown adipocytes (ciBAs) from the primary culture of human dermal fibroblasts in a transgene- and serum-free manner [27,28]. In human BAT, FAs hydrolyzed from intracellular triglycerides are known to be a predominant fuel source for heat production [29]. We harnessed the serum-free medium to examine the impact of FA depletion, resembling a lean condition on UCP1 expression and mitochondrial-energy status in ciBAs. This study provides insights into the regulation of *UCP1* gene expression in a human-brown-adipocyte model under diverse nutritional conditions, which would be useful in identifying drug targets for the treatment of obesity.

## 2. Materials and Methods

### 2.1. Cell Culture

All human dermal fibroblasts were purchased from DS Pharma Biomedical Co. (Osaka, Japan). Fibroblasts derived from a human subject, aged 38 years (HDF38), were used unless otherwise indicated. The information regarding human subjects HDF38, HDF35, HDF37, HDF44, and HDF54 is listed in Table S3. Approximately 1.5 × 10^5^ cells were seeded on a 35-mm dish with high-glucose Dulbecco’s Modified Eagle Medium (DMEM; 11995-065; Gibco, Palo Alto, CA, USA), supplemented with 10% fetal bovine serum (FBS; SH30088.03; HyClone, Logan, UT, USA) and penicillin/streptomycin (Gibco). When the cells reached 80–90% confluence, the medium was changed to start the direct conversion into ciBAs, using the serum-free brown adipogenic medium (SFBAM) prepared as reported previously [28]. The SFBAM was supplemented with either linoleic acid (LA)- and oleic acid (OA)-bovine serum albumin (FA BSA; L9655-5ML; Sigma-Aldrich, Saint Louis, MO, USA) or FA-free BSA (017-15141; FUJIFILM Wako, Osaka, Japan). The human fibroblasts were converted into ciBAs using SFBAM, including the chemical cocktail, designated as RoFB, consisting of 1 μM Rosiglitazone (184-02651; FUJIFILM Wako), 7.5 μM Forskolin (063-02193; FUJIFILM Wako), and 20 ng/mL human recombinant bone morphogenetic protein 7 (BMP7; 026-19171; FUJIFILM Wako) for 3 weeks, unless otherwise indicated. Carnitine (030-11353; FUJIFILM Wako), palmitic acid (PA; 165-00102; FUJIFILM Wako), OA (155-03401; FUJIFILM Wako), LA (126-03612; FUJIFILM Wako), α-linolenic acid (ALA; 127-05901; FUJIFILM Wako), eicosapentaenoic acid (90110; Cayman Chemical, Ann Arbor, MI, USA), docosahexaenoic acid (90310; Cayman Chemical), etomoxir (11969; Cayman Chemical), and capsaicin (030-11353; FUJIFILM Wako) were used during the conversion at different concentrations, as indicated. ciBAs were transiently treated with mitochondrial complex inhibitors, oligomycin A (75351; Sigma-Aldrich), and FCCP (C3463; Tokyo Chemical Industry, Tokyo, Japan).

The immortalized preadipocyte cell line (hTERT A41hBAT-SVF), isolated from human deep-neck fat tissue, was purchased from the American Type Culture Collection (CRL-3385; ATCC, VA, USA). The A41BAT-SVF cells were cultured and differentiated in the same manner as human dermal fibroblasts and ciBAs. Human MSCs derived from adipose tissue (AdMSCs) and bone marrow (BmMSCs) (Table S3) were purchased from TaKaRa Bio (C-12977 and C-12974; TaKaRa Bio, Shiga, Japan). These cells were maintained and expanded using Mesenchymal Stem Cell Growth Medium 2 (C-28009; TaKaRa Bio). When the cells reached 80–90% confluence, AdMSCs and BmMSCs were differentiated into mature adipocytes in the same manner as ciBAs. These commercial human cells have been approved for in vitro research use only. All experimental procedures for cell cultures were conducted according to the general guidelines of Kyoto Prefectural University of Medicine.

### 2.2. Gene Expression Analysis

Total RNA was extracted from the control cells, ciBAs, A41BAT-SVF cells, and MSCs cultured under different experimental conditions using FastGene RNA Basic Kit (FG-80250; Nippon Genetics, Tokyo, Japan). After reverse transcription using ReverTra Ace qPCR RT Master Mix with gDNA Remover (FSQ-301; TOYOBO, Osaka, Japan), real-time PCR analysis was performed using Power SYBR Green PCR Master Mix (4367659; Thermo Fisher Scientific, Waltham, MA, USA). The reactions were carried out in triplicate and under the following conditions: 10 min at 95 °C, followed by 40 cycles for 15 s at 95 °C and 60 s at 60 °C. All the results were normalized to the amount of *TBP* mRNA. The ratio of *UCP1* to *FABP4* mRNA was calculated as an indicator for adipocyte browning. All the primers for quantitative reverse transcription-polymerase chain reaction (qRT-PCR) were designed to span exon–exon junctions. Primer-BLAST (https://www.ncbi.nlm.nih.gov/tools/primer-blast/, last accessed on 5 April 2021) was performed to verify the absence of possible non-specific amplifications. Unless otherwise indicated, the average of the three biological replicates was calculated. All primer sequences for qRT-PCR are listed in Table S4.

### 2.3. Immunoblotting

Total proteins were extracted from the control fibroblasts, ciBAs, and A41BAT-SVF cells using RIPA Buffer (182-02451; FUJIFILM Wako), supplemented with Phosphatase Inhibitor Cocktail Solution II (160-24371; FUJIFILM Wako) and Protease Inhibitor Cocktail Set I (165-256021; FUJIFILM Wako). The extracted proteins were subjected to sodium dodecyl sulfate-polyacrylamide gel electrophoresis (SDS-PAGE) using a 10% polyacrylamide gels concentration and then transferred on to polyvinylidene fluoride (PVDF) membranes (LC2005; Thermo Fisher Scientific). The membranes were blocked with 3% skim milk followed by incubation with antibodies against UCP1 (1:1000 dilution; MAB6158; R&D Systems, MN, USA) and β-Actin (1:10,000 dilution; A5316; Sigma-Aldrich) at 4 °C overnight. The membranes were incubated with goat horseradish peroxidase (HRP)-conjugated anti-mouse antibody (1:15,000 dilution; sc-2005; Santa Cruz Biotechnology, CA, USA) for 1 h at room temperature. Immunoreactive bands were detected using Immobilon Western Chemiluminescent HRP Substrate (WBKLS0500; Merck Millipore, Darmstadt, Germany). The intensity of each band was quantified by densitometry using ImageJ software (version 1.52; National Institutes of Health, Bethesda, MD, USA) [30]. β-Actin was used as a loading control for normalization. The experiments were performed independently at least twice. The full-length Western blots are shown in Figure S8.

### 2.4. Transcriptome Analysis

For RNA-sequencing (RNA-Seq) analysis, using FastGene RNA Premium Kit (FG-81050; Nippon Genetics), total RNA was prepared from three biological replicates of the control fibroblasts (HDF38) and ciBAs treated with FA-free BSA, which were referred to as NoC(FA-free) and RoFB(FA-free), respectively. Total RNA was also extracted from three biological replicates of ciBAs treated with either carnitine or PA, denoted as RoFB(FA) + Car and RoFB(FA-free) + PA, respectively. RNA integrity number values were over 9 in all the tested RNA samples. The library was prepared using TruSeq stranded mRNA Library Prep Kit (RS-122-2101; Illumina, CA, USA), in accordance with the low sample (LS) protocol of the manufacturer. Sequence data were generated using 100 bp paired-end sequencing on NovaSeq 6000 System (Illumina). Trimmed reads were mapped to a reference genome (NCBI GRCh37) with HISAT2. After read mapping, StringTie was used for transcript assembly. Thereafter, gene/transcript abundance was calculated from the read counts and normalized as fragments per kilobase of transcript per million mapped sequence reads (FPKM). For the identification of DEGs, statistical analysis was performed using fold change and exact test using edgeR per comparison pair. Significant results satisfying the conditions of |fold change| ≥ 2 and the exact test *p*-value < 0.05 were selected. If more than one read count value was zero, it was not included in the analysis. The RNA-Sequencing data in this study have been deposited in the DNA Data Bank of Japan (DDBJ) Sequenced Read Archive (https://www.ddbj.nig.ac.jp/dra/index-e.html, last accessed on 20 May 2022) under the accession numbers DRA014166, DRA014311, and DRA014312. Heat maps were generated using Heatmapper (http://www.heatmapper.ca/, last accessed on 30 November 2021) [31]. Hierarchical clustering analysis was based on Euclidean distance. Each row represents a gene, and each column represents z-scored FPKM of each sample. The green and magenta gradients represent lower and higher gene expression, respectively. GO-term-enrichment analysis was performed using the DAVID Bioinformatics Resources 6.8 (https://david.ncifcrf.gov/, last accessed on 30 November 2021) [32].

### 2.5. Oxygen Consumption Rate (OCR)

Human dermal fibroblasts and A41BAT-SVF preadipocytes were seeded on a 96-well plate. These cells were converted into brown-like adipocytes for three weeks, as described above. Before the measurement, the cells were washed and incubated with non-buffered DMEM supplemented with 25 mM glucose, 2 mM glutamine, and 1 mM pyruvate at 37 °C in a non-CO_2_ incubator for 1 h. Thereafter, OCR was then measured using Seahorse XF96 Extracellular Flux Analyzer (Agilent Technologies Inc., Santa Clara, CA, USA) and the Seahorse XF Cell Mito Stress Test Kit (103015-100; Agilent Technologies Inc.) in accordance with the instructions of the manufacturer. In brief, oligomycin, FCCP, and antimycin A/rotenone were added into each well via an injection apparatus to reach final concentrations of 2 μM, 0.3 μM, and 0.5 μM, respectively. Extracellular-acidification rate (ECAR) was simultaneously measured in each experiment. The mitochondria-dependent OCRs corresponding to each parameter were determined by subtracting antimycin A/rotenone-insensitive OCR values from the other OCR values. The experiments were performed independently twice.

### 2.6. Measurement of Mitochondrial Membrane Potential (MMP)

MMP in the control fibroblasts and ciBAs were evaluated using MT-1 MitoMP Detection kit (MT13; Dojindo, Kumamoto, Japan). After washing twice with phosphate-buffered saline (PBS), the cells were treated with the MT-1 dye for 30 min at 37 °C, in accordance with the instructions of the manufacturer. Then, the cells were washed twice with PBS and fixed with 4% paraformaldehyde for 10 min. Next, images were taken using BZ-X710-All-in-One Fluorescence Microscope (Keyence, Osaka, Japan) with a 20× objective lens (CFI Plan Fluor 20X, Nikon, Tokyo, Japan). The scale bars represent 100 μm. The area of the staining was quantified using ImageJ software from at least six different optical sections. The experiments were performed independently twice.

### 2.7. Measurement of Triglyceride Contents and Glycerol-3-Phosphate Dehydrogenase (GPDH) Activity

Cellular triglycerides were solubilized from the control fibroblasts and ciBAs by heating at 90 °C in 5% NP-40 substitute (145-09701; FUJIFILM Wako). Next, triglyceride contents were determined using Triglyceride Assay kit (Ab65336; Abcam, Cambridge, UK), in accordance with the instructions of the manufacturer. For GPDH activity, cell lysates extracted from the control fibroblasts and ciBAs were measured using GPDH Assay kit (AK01; Cosmo Bio Co., Ltd., Tokyo, Japan), in accordance with the instructions of the manufacturer. All the experiments were performed in triplicates. The triglyceride contents and GPDH activity were normalized to protein levels in each sample.

### 2.8. Quantification and Statistical Analysis

All the results are presented as the mean ± the standard deviation (SD) or the standard error of the mean (SEM). “*n*” represents the number of biological replicates. Statistical analyses were performed by a two-tailed Student’s *t*-test using the Excel (Microsoft) program. Statistically significant differences are annotated as follows: * *p* < 0.05, ** *p* < 0.05, and *** *p* < 0.001.

## 3. Results

### 3.1. Fatty-Acid Depletion Induces UCP1 Expression in ciBAs

**Figure 1:**
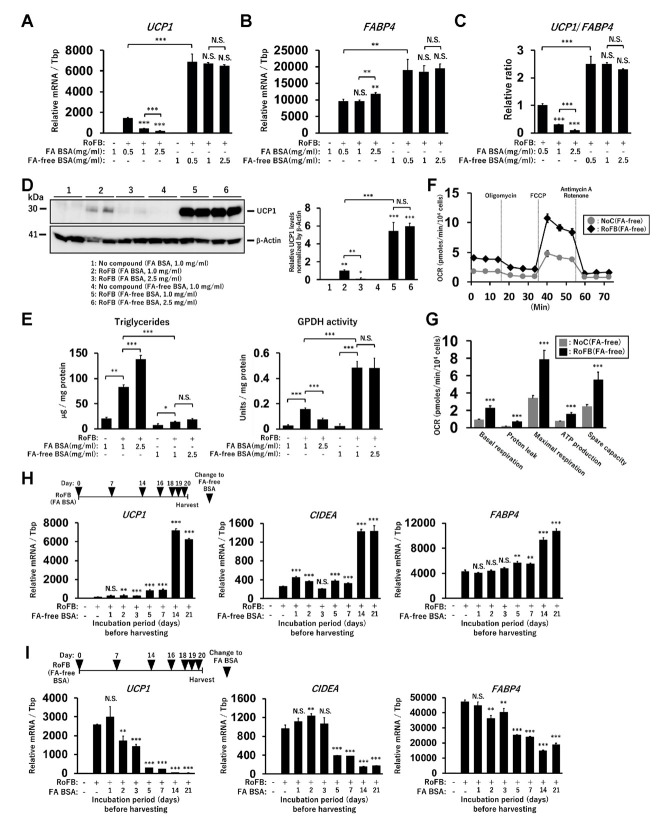
Induction of *UCP1* expression in ciBAs converted under FA-free conditions. (**A**,**B**) The expression of *UCP1* (**A**) and *FABP4* (**B**) mRNA was quantified using qRT-PCR in ciBAs converted by different amounts of either FA-binding BSA (FA BSA) or FA-free BSA for 3 weeks. (**C**) The ratio of *UCP1* to *FABP4* expression was calculated to evaluate adipocyte browning. (**D**) UCP1 protein levels were quantified by immunoblotting. The band intensities were measured by densitometry using ImageJ software. (**E**) Cellular triglyceride content and glycerol-3-phosphate dehydrogenase 1 (GPDH) activity were measured in ciBAs. (**F**) Oxygen consumption rate (OCR) was measured in the control fibroblasts (grey circles) and ciBAs (black diamonds) converted under the FA-free condition using the flux analyzer. Oligomycin, FCCP, and antimycin A/Rotenone were added during the measurement as indicated. (**G**) Each OCR corresponding to basal respiration, proton leak, maximal respiration, ATP production, and spare respiratory capacity was calculated. (**H**,**I**) The expression of *UCP1*, *CIDEA*, and *FABP4* was measured in ciBAs treated with FA-free BSA or FA BSA for different incubation periods (days), before harvesting the cells on day 21. The timing of BSA replacement is shown in the illustration. Data represent mean ± SD (*n* = 3). Student’s *t*-test: * *p* < 0.05, ** *p* < 0.01, *** *p* < 0.001, N.S.; not significant.

The direct conversion of human dermal fibroblasts into ciBAs was performed using the serum-free brown adipogenic medium, including a chemical cocktail of Rosiglitazone, Forskolin, and BMP7, designated as (RoFB) hereafter [28]. To investigate the impact of the loss of FAs in the medium on the conversion, FA-free BSA was administered, instead of LA- and OA-binding BSA (FA BSA). During the conversion period of up to five weeks, cells with a round morphology different from flat and spindle-shaped fibroblasts gradually increased (Figure S1A). The expression of several adipocyte marker genes, such as *UCP1*, *CKMT1*, *CITED1*, and *FABP4*, was increased during the conversion (Figure S1B), indicating that the round cells with sparse lipid droplets were ciBA-like brown adipocytes. Next, the fibroblasts were converted in the medium containing different amounts of FA BSA and FA-free BSA for three weeks (Figure 1A). The increased amounts of FA BSA reduced *UCP1* mRNA levels in a dose-dependent manner. In contrast, *UCP1* expression was induced regardless of the amount of FA-free BSA. The expression of *FABP4* was moderately increased during FA-free BSA treatment (Figure 1B). The ratio of *UCP1* to *FABP4* expression levels indicated that adipocyte browning might be suppressed in the presence of FAs (Figure 1C). The induced UCP1 protein levels reflected mRNA levels in ciBAs treated with FA-free BSA (Figure 1D). Other lines of human dermal fibroblasts derived from different subjects aged 35, 37, 44, and 54 years exhibited induction of *UCP1* expression through treatment with FA-free BSA (Figure S1C). 

Intracellular triglyceride levels were repressed in ciBAs converted in the FA-free conditions (Figure 1E). Glycerol-3-phosphate dehydrogenase (GPDH), a rate-limiting enzyme for triglyceride synthesis, was inversely correlated with the accumulation of triglycerides in ciBAs. ciBAs converted in the FA-free condition enhanced the oxygen-consumption rate (OCR) compared to that in the control cells (Figure 1F). OCR corresponding to basal respiration, proton leak, maximal respiration, ATP production, and spare respiratory capacity was increased (Figure 1G). Next, we estimated the incubation period required with either FA BSA or FA-free BSA to observe *UCP1* gene expression alterations. FA BSA was replaced with FA-free BSA during the conversion for the indicated incubation periods, before harvesting on day 21 (Figure 1H). The expression of *UCP1*, *CIDEA*, and *FABP4* was gradually increased during the treatment with FA-free BSA, particularly for more than 5 days; however, the treatment with FA BSA instead of FA-free BSA strongly reduced the expression (Figure 1I). These results indicated that a relatively prolonged incubation period was required to vary *UCP1* expression by altering the amount of FA in the medium.

### 3.2. Treatment with Free FAs Reduces UCP1 Expression in ciBAs

**Figure 2:**
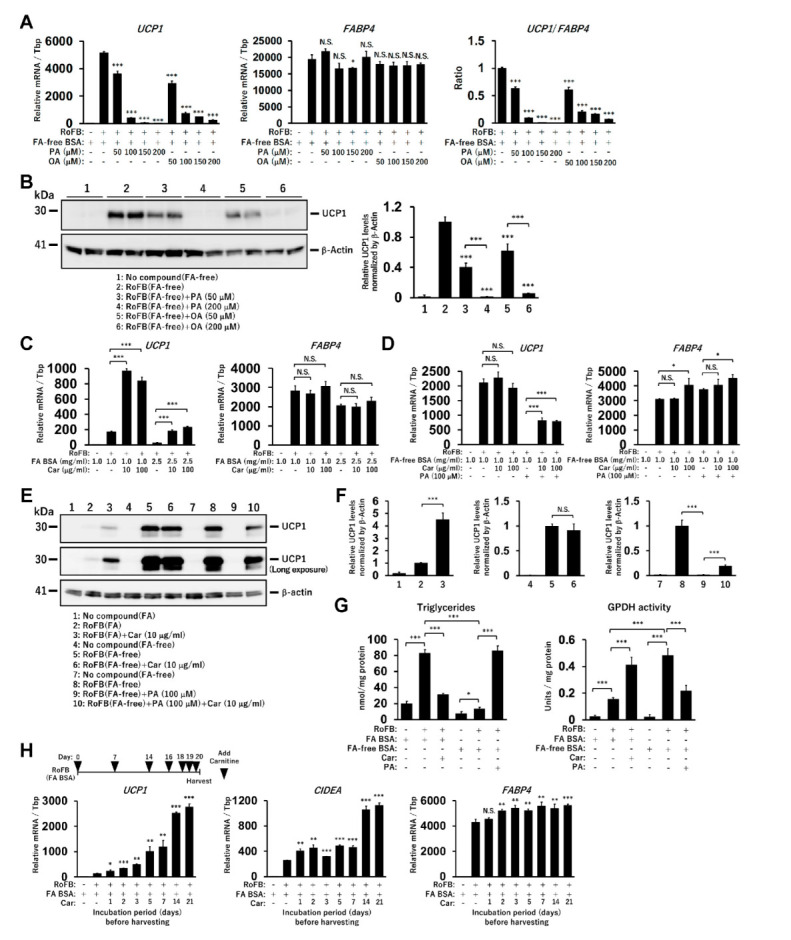
Treatment with FAs and carnitine modulates *UCP1* expression in ciBAs. (**A**) The expression of *UCP1* and *FABP4* mRNA and the ratio were quantified using qRT-PCR in ciBAs treated with either palmitic acid (PA) or oleic acid (OA) at different concentrations as indicated. (**B**) UCP1 protein levels were quantified using immunoblotting. (**C**,**D**) The expression of *UCP1* and *FABP4* mRNA was quantified using qRT-PCR in ciBAs treated with carnitine (10 and 100 μg/mL) and FA BSA (**C**) or FA-free BSA (**D**). (**E**) The UCP1 and β-Actin proteins were immunoblotted in ciBAs converted under different conditions, as indicated. (**F**) The relative UCP1 protein levels were calculated from band intensities shown in Figure S2C. (**G**) Cellular triglyceride content and GPDH activity were measured in ciBAs treated with either carnitine or PA. (**H**) The expression of *UCP1*, *CIDEA*, and *FABP4* was measured using qRT-PCR in ciBAs treated with FA BSA and carnitine (10 μg/mL) for different incubation periods (days), before harvesting the cells on day 21. The illustration represents the timing of the addition of carnitine. Data represent mean ± SD (*n* = 3). Student’s *t*-test: * *p* < 0.05, ** *p* < 0.01, *** *p* < 0.001, N.S.; not significant.

To further confirm whether the supply of free FAs repressed *UCP1* expression in ciBAs, either PA or OA was administered at different concentrations (50–200 µM) in the presence of FA-free BSA. *UCP1* mRNA levels were strongly reduced by the treatment in a dose-dependent manner (Figure 2A), whereas *FABP4* expression remained largely unchanged. The ratio of *UCP1* to *FABP4* mRNA levels indicated that adipocyte browning might be repressed in ciBAs. Accordingly, UCP1 protein levels were also repressed (Figure 2B). In addition, the treatment with polyunsaturated fatty acids, either LA or ALA, repressed *UCP1* expression but not *FABP4* (Figure S2A). The treatment with either eicosapentaenoic acid or docosahexaenoic acid also repressed *UCP1* expression in a dose-dependent manner (Figure S2B). These results suggested that the prolonged treatment with any of these FAs commonly reduced *UCP1* expression in ciBAs.

### 3.3. Treatment with Carnitine Induces UCP1 Expression in ciBAs

To evaluate the effect of accelerated FA oxidation in ciBAs, carnitine, which transfers long-chain FAs from the cytoplasm into mitochondria, was administered. The continuous treatment with carnitine increased the expression of *UCP1*, but not *FABP4*, in the presence of FA-binding BSA (Figure 2C). In contrast, *UCP1* expression was almost unchanged by carnitine in the presence of FA-free BSA (Figure 2D). The repression caused by PA treatment was partially reversed by carnitine, suggesting that its effect was dependent on the presence of FAs. The UCP1 protein levels reflected these changes in mRNA levels (Figure 2E,F and Figure S2C). The treatment with carnitine and PA decreased and increased intracellular triglycerides contents, respectively, whereas GPDH activity was inversely regulated by carnitine as well as PA treatments (Figure 2G and Figure S2D). The results suggested that the prolonged treatment with carnitine consumed the cellular triglycerides stored in ciBAs. Accordingly, longer incubation periods with carnitine gradually enhanced the expression of *UCP1* and *CIDEA* (Figure 2H). These mRNAs were also increased by carnitine under the treatment with PA and FA-free BSA (Figure S2E). Furthermore, etomoxir, an inhibitor of carnitine palmitoyltransferase 1a, repressed the expression of *UCP1* and *CIDEA* a few days after the treatment (Figure S2F).

### 3.4. FA-Depleted Conditions Enhance the Expression of Metabolic Genes in ciBAs

**Figure 3:**
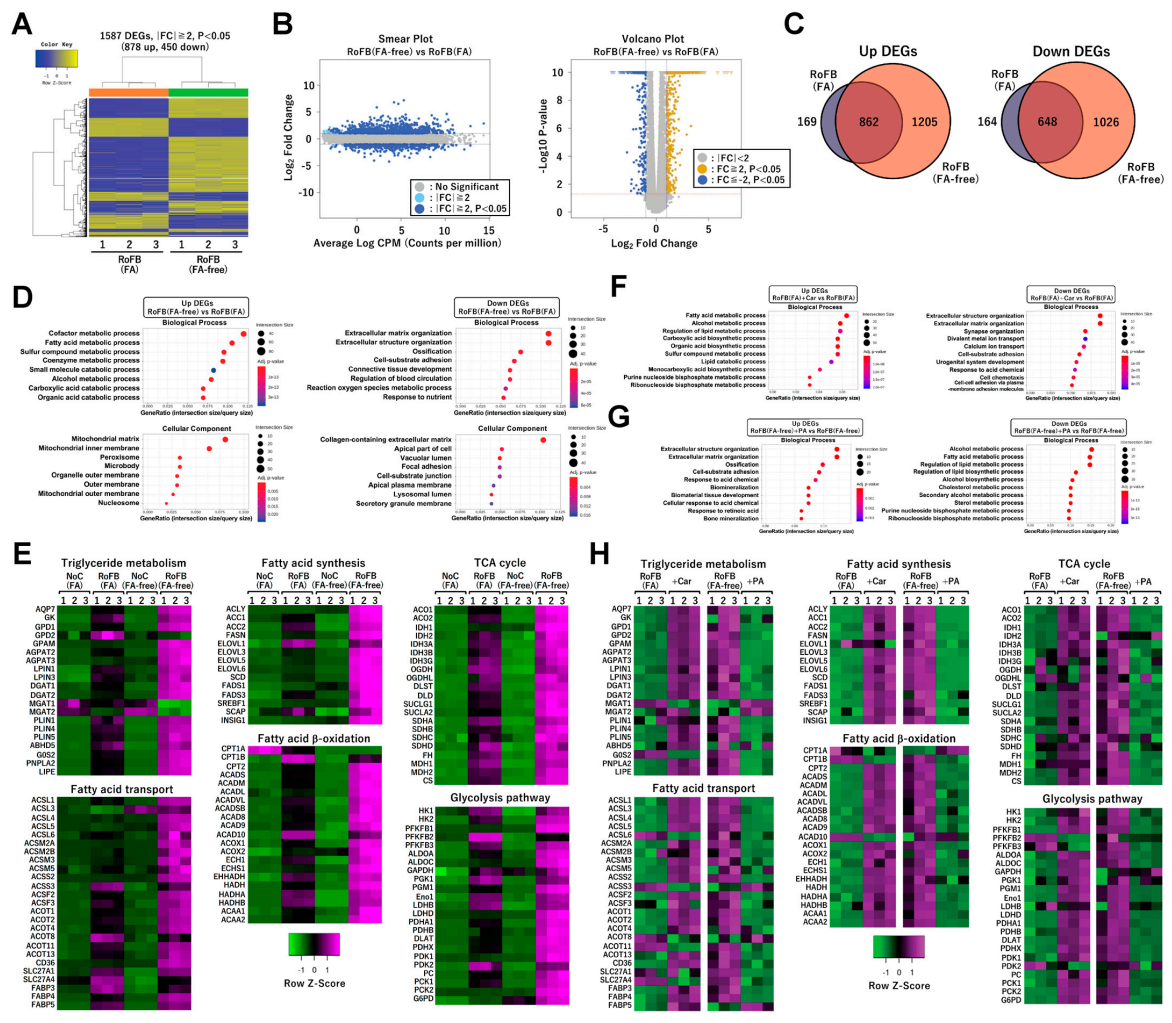
FA depletion activates a broad range of lipid metabolic genes in ciBAs. (**A**) Heat-map and hierarchical clustering analysis represent 1587 differentially expressed genes (DEGs) |fold change| ≥ 2, *p* < 0.05) between ciBAs, RoFB(FA-free), and RoFB(FA). (**B**) Smear and Volcano plots represent logarithmic fold change, *p*-value, and counts per million obtained from the comparison between RoFB(FA-free) and RoFB(FA). (**C**) Venn diagrams represent the overlap of upregulated and downregulated DEGs between RoFB(FA-free) and RoFB(FA). (**D**) Gene ontology (GO)-term enrichment analysis was performed in the upregulated and downregulated DEGs. The top 10 GO terms are represented in the categories of biological process and cellular component. (**E**) A heat-map analysis shows z-scored fragments per kilobase of transcript per million mapped sequence reads (FPKM) obtained from the RNA-Seq data in each functional category, as indicated. The color scale represents mRNA levels of each gene in green (lower expression) and magenta (higher expression). (**F**,**G**) GO analysis was performed in the upregulated and downregulated DEGs between RoFB(FA) and RoFB(FA) + Car (**F**) and between RoFB(FA-free) and RoFB(FA-free) + PA (**G**). The top 10 GO terms are represented in the categories of biological process. (**H**) Heat maps show z-scored FPKM obtained from the RNA-Seq results between RoFB(FA) and RoFB(FA) + Car and between RoFB(FA-free) and RoFB(FA-free) + PA in each functional category.

RNA-sequencing (RNA-Seq) analysis was performed to characterize the transcriptional changes in ciBAs converted in the FA-free conditions. The analysis between ciBAs and control fibroblasts treated with FA-free BSA, RoFB(FA-free), and NoC(FA-free) detected 2067 upregulated and 1674 downregulated differentially expressed genes (DEGs) (Figure S3A). Gene ontology (GO)-enrichment analysis indicated that the upregulated and downregulated DEGs were functionally related to energy metabolism and extracellular matrix, respectively (Figure S3B,C), which was similar to our previous results in ciBAs treated with FA BSA and RoFB(FA) [33]. Next, the comparison between RoFB(FA-free) and RoFB(FA) ciBAs detected 878 upregulated and 450 downregulated DEGs (Figure 3A). The smear and volcano plots showed that these DEGs with over two-fold changes (FCs) were properly distributed with widespread CPM (counts per million) and *p*-values (Figure 3B). Venn diagrams represented that the upregulated and downregulated DEGs were overlapped between these ciBAs (Figure 3C). GO-enrichment analysis suggested that the upregulated DEGs were categorized into functional groups, such as FA metabolism and mitochondria, whereas the downregulated DEGs were related to the extracellular matrix (Figure 3D). Heat-map analysis represented that the expression of a series of metabolic genes involved in the triglyceride metabolism, FA β-oxidation, FA synthesis, FA transport, tricarboxylic acid (TCA) cycle, and glycolysis was enhanced in RoFB(FA-free) to a greater extent than in RoFB(FA) (Figure 3E). Furthermore, the expression of mitochondria-related genes involved in electron-transfer-chain (ETC) complex, adaptive thermogenesis, and extracellular matrix, was more varied in RoFB(FA-free) (Figure S3D).

Transcriptome analysis was also performed in ciBAs treated with either carnitine or PA (Figure S4A,B), which were referred to as RoFB(FA) + Car and RoFB(FA-free) + PA, respectively. In the comparison between RoFB(FA) and RoFB (FA) + Car, GO analysis indicated that carnitine enhanced and repressed FA metabolic genes and extracellular matrix-related genes, respectively (Figure 3F). In contrast, PA treatment inversely regulated the transcription of these genes involved in FA metabolism and extracellular matrix (Figure 3G). Heat-map analysis showed opposite effects between ciBAs treated with carnitine and PA (Figure 3H and Figure S4C). 

### 3.5. FA-Depleted Conditions Activate a Set of Lipid Metabolic Genes along with UCP1

**Figure 4:**
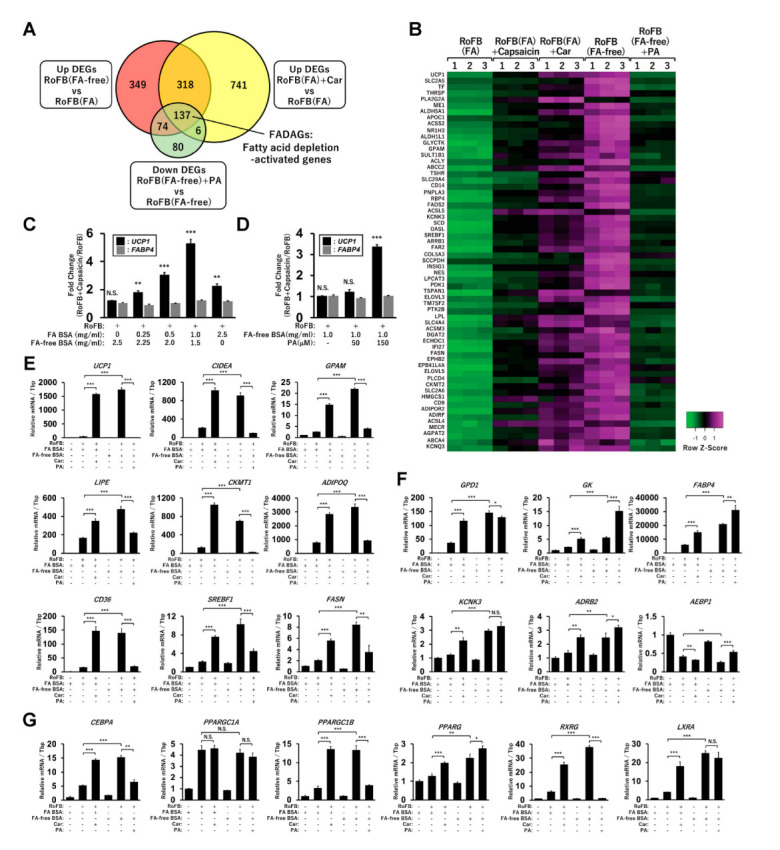
A set of lipid metabolic and synthetic genes are regulated with *UCP1* in ciBAs. (**A**) Venn diagram represents the overlap of the upregulated DEGs in RoFB(FA-free) and RoFB(FA) + Car and the downregulated DEGs in RoFB(FA-free) + PA. The 137 overlapped DEGs are referred to as fatty acid depletion-activated genes (FADAGs). (**B**) Heat map shows transcriptional profiles of representative FADAGs in ciBAs converted under different conditions. (**C**) The fold change of *UCP1* and *FABP4* expression in the treatment with capsaicin was quantified in ciBAs treated with varying amounts of FA-binding BSA. (**D**) The fold change was quantified in ciBAs treated with FA-free BSA (1.0 mg/mL) and PA (50 and 150 µM). (**E**,**F**) The expression of *UCP1*, *CIDEA*, *GPAM*, *LIPE*, *CKMT1*, *ADIPOQ*, *CD36*, *SREBF1*, and *FASN* (**E**) as well as *GPD1*, *GK*, *FABP4*, *KCNK3*, *ADBR2*, and *AEBP1* (**F**) was quantified using qRT-PCR in ciBAs converted under different conditions. (**G**) The expression of transcription factors, such as *CEBPA*, *PPARGC1A*, *PPARGC1B*, *PPARG*, *RXRG*, and *LXRA* was quantified. Data represent mean ± SD (*n* = 3). Student’s *t*-test: * *p* < 0.05, ** *p* < 0.01, *** *p* < 0.001, N.S.; not significant.

To precisely identify a gene set regulated along with *UCP1*, the upregulated DEGs in RoFB(FA-free) and RoFB(FA) + Car and the downregulated DEGs in RoFB(FA-free) + PA were compared (Figure 4A). Venn diagram analysis identified 137 genes, denoted as fatty-acid-depletion activated genes (FADAGs) (Table S1). The functions of FADAGs were mainly categorized into lipid-metabolic and synthetic processes (Table S2). The expression levels of *UCP1* and representative FADAGs were increased in the treatment with carnitine and FA-free BSA, whereas PA treatment repressed the expression in a coordinated manner (Figure 4B), indicating that UCP1 function might be closely associated with lipid-metabolic and synthetic pathways. We have recently reported that the treatment with capsaicin directly promoted the browning of ciBAs and *UCP1* expression [34]. Notably, as presented in the heat map in Figure 4B, capsaicin also activated the transcription of FADAGs. To clarify the dependency of capsaicin on FAs in the medium, the induction of UCP1 by capsaicin was evaluated in ciBAs treated with different amounts of FA-binding BSA (Figure 4C). The fold changes of *UCP1* mRNA by capsaicin increased approximately five times in the presence of FA BSA at 1.0 mg/mL concentration. In contrast, the treatment with FA-free BSA produced almost no effect by capsaicin on *UCP1* activation. However, additional treatment with PA (150 μM) recovered the induction by capsaicin approximately three times (Figure 4D). Moreover, capsaicin activation was inhibited by the treatment with either carnitine or etomoxir (Figure S5A), suggesting that the effects were dependent on extracellular FAs and cellular FA metabolism. 

qRT-PCR analysis was performed to confirm the transcriptional changes of lipid metabolic genes observed in the RNA-Seq results. The expression of *CIDEA*, *GPAM*, *LIPE*, *CKMT1*, *ADIPOQ*, *CD36*, *SREBF1*, and *FASN* was repressed by the treatment with either FA BSA or PA, whereas the expression was increased in the treatment with either carnitine or FA-free BSA. This finding indicated that these genes were transcriptionally regulated in a similar manner to that of *UCP1* (Figure 4E). The expression of *GPD1*, *GK*, *FABP4*, *KCNK3*, and *ADBR2* was elevated in the treatment with either carnitine or FA-free BSA; however, it was not repressed by PA treatment (Figure 4F). The expression of *AEBP1*, a transcriptional repressor for adipogenesis, was inversely repressed in the FA-depleted conditions. The expression of *CEBPA*, *PPARGC1B*, *PPARG*, *RXRG*, and *LXRA*, encoding-transcription factors critical for brown adipogenesis, was also activated under the FA-depleted conditions (Figure 4G). However, *PPARGC1A* expression was not regulated under these conditions. The transcriptional changes were largely consistent with corresponding RNA-Seq results (Figure S5B–D). 

### 3.6. Immortalized Human-Brown-Adipocyte Cell Line Shows UCP1 Induction under the FA-Free Condition

**Figure 5:**
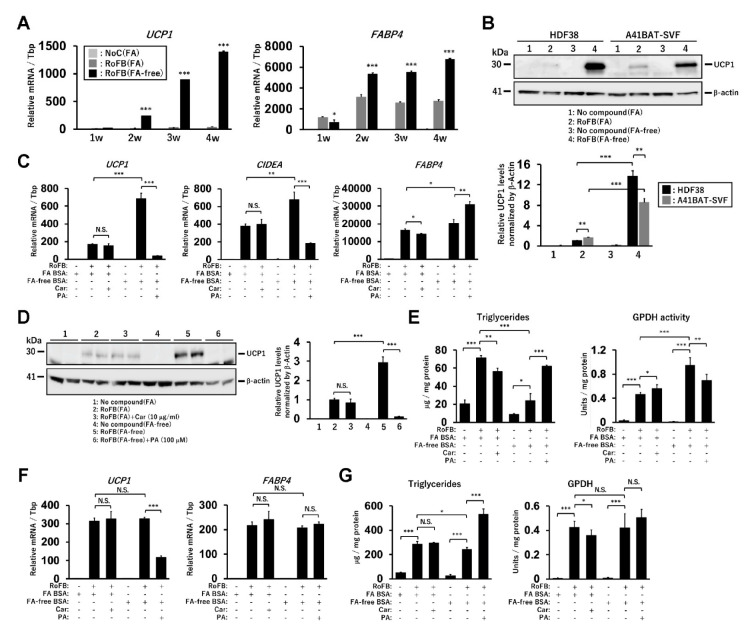
Regulation of *UCP1* expression by FAs in an immortalized human-brown-preadipocyte cell line (hTERT A41BAT-SVF). (**A**) The expression of *UCP1* and *FABP4* was quantified using qRT-PCR during differentiation of A41BAT-SVF preadipocytes using RoFB in the treatment with either FA BSA or FA-free BSA, from 1 to 4 weeks as indicated. (**B**) UCP1 protein levels were compared between ciBAs and A41BAT-SVF adipocytes. (**C**) The expression of *UCP1*, *CIDEA*, and *FABP4* was quantified using qRT-PCR in A41BAT-SVF adipocytes differentiated using carnitine or PA. (**D**) The UCP1 protein was immunoblotted in A41BAT-SVF adipocytes differentiated under various conditions. (**E**) Cellular triglyceride content and GPDH activity were measured in A41BAT-SVF adipocytes. (**F**) The expression of *UCP1* and *FABP4* was quantified using qRT-PCR in adipocytes differentiated from adipose-tissue-derived mesenchymal stem cells (AdMSCs) using carnitine or PA. (**G**) Cellular triglyceride content and GPDH activity were measured in AdMSC-derived adipocytes. Data represent mean ± SD (*n* = 3). Student’s *t*-test: * *p* < 0.05, ** *p* < 0.01, *** *p* < 0.001, N.S.; not significant.

To verify whether UCP1 expression is similarly regulated by the FA depletion in another model of human brown adipocytes, hTERT A41BAT-SVF, an immortalized human-brown-preadipocyte cell line, was examined. A41BAT-SVF cells were differentiated into mature adipocytes using the same protocol as that for ciBAs in the presence or absence of FAs for up to 4 weeks (Figure 5A). Compared to FA-binding BSA treatment, the expression of *UCP1* was strongly induced by FA-free BSA treatment. *FABP4* expression was moderately increased in FA-free BSA treatment, similar to the observations in ciBAs. UCP1 protein was induced in A41BAT-SVF cells treated with FA-free BSA (Figure 5B). The expression levels were comparable or slightly decreased, compared to those in ciBAs. Although the expression of *UCP1* and *CIDEA* was almost unchanged by the treatment with carnitine in A41BAT-SVF adipocytes, the treatment with PA repressed the expression (Figure 5C). UCP1 protein levels reflected the mRNA levels under these conditions (Figure 5D). The expression of lipid metabolic genes and transcriptional factors analyzed in ciBAs was rationally regulated under the FA-free condition and PA treatment (Figure S6A). In A41BAT-SVF adipocytes, triglyceride contents were decreased and increased by the treatment with FA-free BSA and PA, respectively, whereas the GPDH activity was inversely altered (Figure 5E). These results suggested that UCP1 and lipid metabolic genes could be induced by the FA-free condition in A41BAT-SVF cells as well.

We previously reported several characteristic differences between ciBAs and mature adipocytes differentiated from adipose tissue-derived mesenchymal stem cells (AdMSCs), using the same protocol as that for ciBAs [33,34]. The expression of *UCP1* was unaffected by the treatment with carnitine and FA-free BSA in AdMSC-derived adipocytes (Figure 5F). Both triglyceride contents and GPDH activity in AdMSC-derived adipocytes exhibited only slight changes in the treatment with carnitine and FA-free BSA (Figure 5G). In contrast, the treatment with PA increased the triglyceride content, which could be linked to the reduced expression of *UCP1*. Furthermore, adipocytes differentiated from a different line of MSCs derived from the adipose tissue and bone marrow did not show a clear induction of *UCP1* expression by FA-free BSA (Figure S6B,C).

### 3.7. FA-Depleted Conditions Repress Mitochondrial Proton-Leak Activity and Its Proportion in Basal Respiration

**Figure 6:**
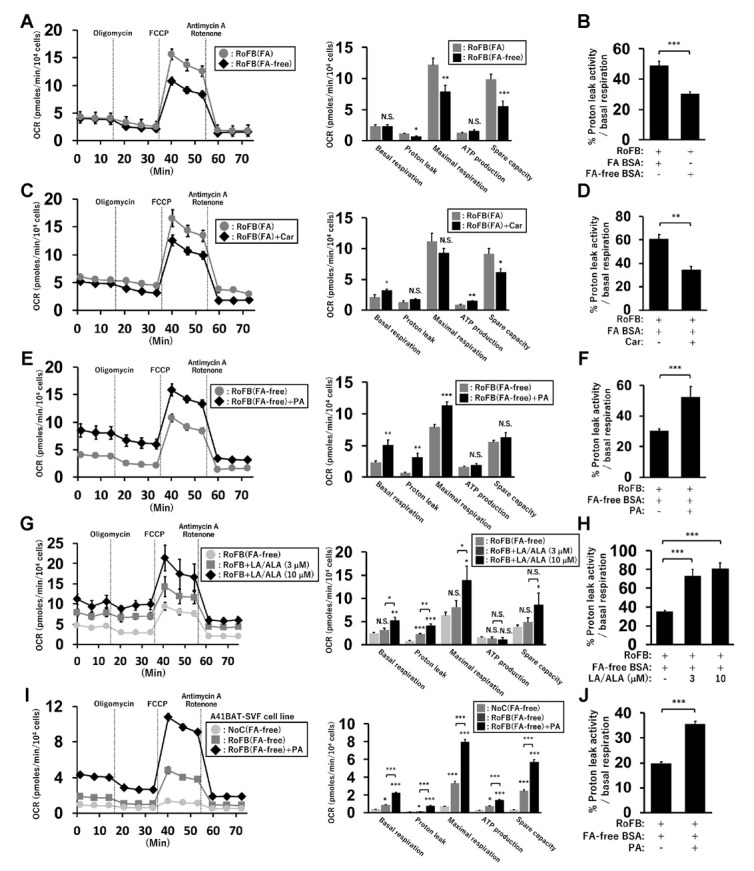
FA supply modulates mitochondrial proton-leak activity in ciBAs. (**A**) Oxygen-consumption rate (OCR) was measured using the flux analyzer in ciBAs, RoFB(FA) (gray circles), and RoFB(FA-free) (black diamonds). Oligomycin, FCCP, and antimycin A/rotenone were added during the measurement as indicated. Each OCR corresponding to basal respiration, proton leak, maximal respiration, ATP production, and spare respiratory capacity was calculated. (**B**) The percent ratio of the proton-leak activity to the basal respiration was compared. (**C**,**D**) OCR (**C**) and the percent ratio (**D**) were compared between RoFB(FA) (gray circles) and RoFB(FA) + Car (black diamonds). (**E**,**F**) OCR (**E**) and the percent ratio (**F**) were compared between RoFB(FA-free) (gray circles) and RoFB(FA-free) + PA (black diamonds). (**G**,**H**) ciBAs were treated with linoleic acid (LA) and α-linolenic acid (ALA) at 3 and 10 μM, respectively. OCR (**G**) and the percent ratio (**H**) were compared in RoFB(FA-free) (light gray circles), RoFB + LA/ALA (3 μM) (gray squares), and RoFB + LA/ALA (10 μM) (black diamonds). (**I**,**J**) OCR (**I**) and the percent ratio (**J**) were compared in the A41BAT-SVF adipocytes differentiated under the same condition as ciBAs, NoC(FA-free) (light gray circles), RoFB(FA-free) (gray squares), and RoFB(FA-free) + PA (black diamonds). Data represent mean ± SEM (*n* = 6–8). Student’s *t*-test: * *p* < 0.05, ** *p* < 0.01, *** *p* < 0.001, N.S.; not significant.

To examine the relationship between *UCP1* expression and mitochondrial-energy status, OCR was compared between ciBAs converted in the presence or absence of FAs (Figure 6A). OCR corresponding to maximal respiration was lowered in RoFB(FA-free) compared to that in RoFB(FA). OCR corresponding to the proton leak was reduced in RoFB(FA-free), despite UCP1 expression being induced. The percent ratio of proton-leak activity to basal respiration was lowered in the FA-free condition (Figure 6B). Furthermore, the treatment with carnitine did not markedly alter OCR in comparison with RoFB(FA) (Figure 6C). The percent ratio of the proton leak to basal respiration was reduced in RoFB(FA) + Car, similar to the results observed in RoFB(FA-free) (Figure 6D). In contrast, the treatment with PA enhanced OCR corresponding to basal respiration, proton leak, and maximal respiration, but not ATP production and spare capacity (Figure 6E). The percent ratio of the proton leak increased (Figure 6F), resembling the ratio in RoFB(FA). To confirm the effects of other FA treatment, a combination of LA and ALA was administered. All the OCRs, except for that of ATP production, were elevated in a dose-dependent manner (Figure 6G), while the percent ratio of the proton leak increased (Figure 6H). In addition, the A41BAT-SVF-derived adipocytes displayed increased OCRs in the treatment with PA, leading to an increased proportion of proton-leak activity (Figure 6I,J). These results proposed that FA supplies elevated proton-leak activity and the proportion in basal respiration, despite *UCP1* expression being repressed.

### 3.8. FA-Depleted Conditions Are Associated with Low Mitochondrial-Membrane Potential (MMP)

**Figure 7:**
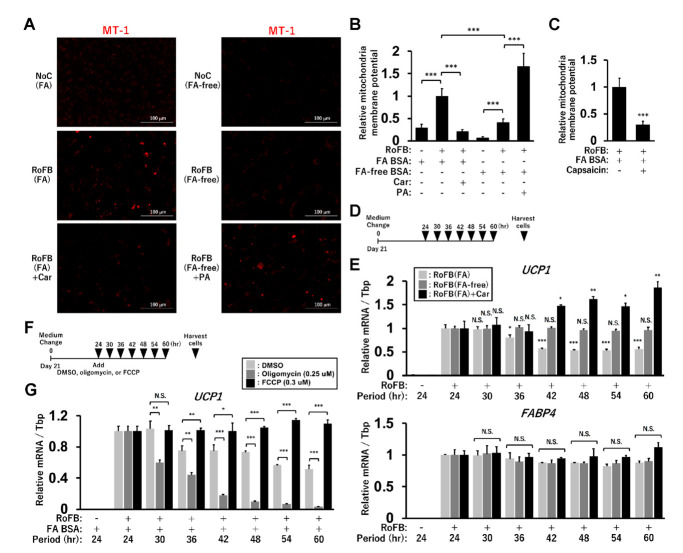
Mitochondrial membrane potential (MMP) affects *UCP1* expression in ciBAs. (**A**) MMP was evaluated by staining with the fluorescent probe, MT-1 dye, in ciBAs cultured under various conditions, as indicated. (**B**,**C**) The area of the staining was quantified using ImageJ software. (**D**) The illustration represents the timing of harvesting ciBAs 24 h after the final medium change on day 21. (**E**) The expression of *UCP1* and *FABP4* was quantified using qRT-PCR in RoFB(FA), RoFB(FA-free), and RoFB(FA) + Car. The expression level in these ciBAs harvested 24 h after the final medium change was normalized to 1. (**F**) The illustration represents the timing of harvesting RoFB(FA) ciBAs after the addition of either DMSO, oligomycin (0.25 µM), or FCCP (0.3 µM). (**G**) The expression of *UCP1* was quantified using qRT-PCR in these ciBAs. The expression level in the ciBAs harvested 24 h after the final medium change was normalized to 1. Data represent mean ± SD (*n* = 3). Student’s *t*-test: * *p* < 0.05, ** *p* < 0.01, *** *p* < 0.001, N.S.; not significant.

To pursue the biological significance of *UCP1* expression regulated by FAs, MMP was evaluated by staining with the fluorescent probe, MT-1 dye (Figure 7A). The staining was enhanced in the control fibroblasts and ciBAs cultured with either FA BSA or PA, while it was decreased in the treatment with either FA-free BSA or carnitine. The quantification of the staining areas indicated that the FA-enriched and -depleted conditions decreased and increased MMP, respectively (Figure 7B), which might be correlated with intracellular triglyceride levels in these conditions. In addition, capsaicin treatment also reduced MMP (Figure 7C). Next, to examine how MMP affects *UCP1* expression, ciBAs were harvested every 6 h, 24 h after the final medium change on day 21 as indicated in the illustration (Figure 7D). ciBAs treated with FA BSA exhibited gradual reduction in *UCP1* expression by approximately 50% during the experimental period (Figure 7E). However, the relative expression of *UCP1* was not reduced, but rather increased in ciBAs treated with FA-free BSA or carnitine. *FABP4* expression was not significantly changed in these ciBAs. These results suggested that *UCP1* induction could be maintained in ciBAs with low MMP caused by the FA depletion. To further test the relationship between MMP and *UCP1* expression, mitochondrial respiration inhibitors were administered 24 h after the final medium change on day 21, as indicated in the illustration (Figure 7F). Oligomycin treatment prevented the dissipation of the proton gradient and caused a transient retention of MMP (Figure S7A). Under this condition, *UCP1* expression was strongly repressed during the experimental period (Figure 7G). Treatment with carbonyl cyanide 4-(trifluoromethoxy)phenylhydrazone (FCCP) reduced MMP (Figure S7B), which could lead to maintenance of *UCP1* expression. These results suggested that MMP status was negatively related to UCP1 expression.

## 4. Discussion

**Figure 8:**
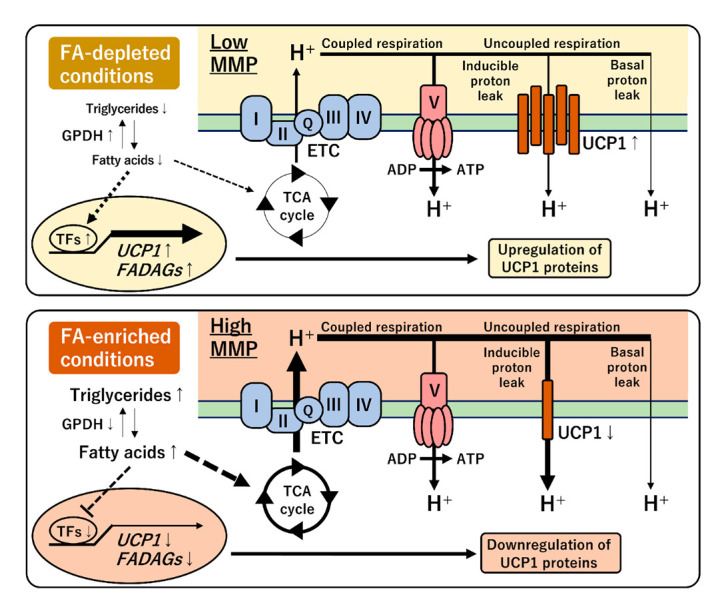
Schematic model of the regulation of UCP1 expression under different FA conditions. The FA-depleted conditions induce UCP1 expression under low MMP (**top panel**). Conversely, the FA-enriched conditions activate MMP, however, UCP1 expression is repressed (**bottom panel**). This feedback regulation between UCP1 expression and mitochondrial-energy status may be required to coordinate the inducible proton-leak activity in human brown adipocytes.

A proton gradient across the mitochondrial inner membrane is driven by FAs, acting as the primary energy source, through the ETC in human brown fat [29,35]. Coupled respiration produces ATP by mediating the ATP synthase, while uncoupled respiration occurs via two types of proton leaks. Basal proton leak is constitutive and uncontrolled; however, inducible proton leak is uniquely regulated by UCP1 expression for subsequent heat generation in brown adipocytes. This study proposed a feedback regulation between UCP1 gene expression and mitochondrial-energy status in a human-brown-adipocyte model (Figure 8). The chronic treatment with FA-free BSA or carnitine depleted intracellular lipid storage and reduced MMP. OCR measurements indicated that the low MMP decreased the proton-leak activity and the proportion in the basal respiration. In the FA-depleted conditions, UCP1 expression was maintained at a high level, suggesting that UCP1 might compensate for proton-leak activity under low MMP. Notably, UCP1 expression activated by capsaicin treatment was also associated with extracellular FA and low MMP. Genome-wide transcriptional analysis indicated that transcription factors, such as CEBPA, PPARGC1B, PPARG, RXRG, and LXRA, might be involved in the expression of UCP1 gene and FADAGs, such as GPAM, ACLY, FADS2, SCD, SREBF1, INSIG1, ELOVL3, DGAT2, and FASN genes (Figure 4). These findings indicate that UCP1 expression is closely connected with FA and triglyceride synthesis pathways. *UCP1* transcription is known to be directly regulated by multiple transcription factors and nuclear receptors [34]. Especially, the expression pattern of *CEBPA*, *PAPRGC1B*, and *RXRG* under different FA conditions was similar to that of *UCP1*, implying their direct role in the regulation of *UCP1* transcription. In contrast, FA administration activated both MMP and proton-leak activity. In the FA-enriched conditions, UCP1 expression was repressed likely to avoid an excessive amount of inducible proton leak under high MMP. This feedback regulation was supported by the perturbation of MMP using oligomycin and FCCP [36,37]. These results suggested that a unique regulatory mechanism underlying UCP1 expression and mitochondrial-energy status was altered by FA availability in human brown adipocytes.

The regulation of UCP1 expression by the FA depletion was also confirmed in the immortalized human brown preadipocyte cell line, A41BAT-SVF, but not in mesenchymal stem-cell-derived adipocytes (Figure 5). The effect of carnitine on UCP1 expression was almost unchanged in the A41BAT-SVF adipocytes, which might be due to the absence of carnitine-induced reduction in the triglyceride content. In contrast, the chronic treatment with FA-free BSA resulted in UCP1 induction along with triglyceride depletion. However, AdMSC-derived adipocytes did not exhibit UCP1 induction in the treatment with carnitine or FA-free BSA. Accordingly, the triglyceride levels were largely unchanged under these conditions, which might be due to sufficient FA biosynthesis to maintain cellular triglycerides in AdMSC-derived adipocytes. PA administration repressed UCP1 expression and led to triglyceride accumulation in both A41BAT-SVF and AdMSC-derived adipocytes. These cell models supported the concept that UCP1 expression was potentially controlled by intracellular triglyceride contents and that the treatment with FAs and carnitine affected UCP1 expression by altering lipid levels. Consistent with this observation, several reports also indicated that human BAT levels were negatively associated with BAT-stored triglyceride content and plasma triglyceride levels during cold stimulation [16,17]. In addition, weight loss after bariatric surgery in morbidly obese women decreased triglyceride content in BAT, which was coupled with the increased proportion of BAT in the supraclavicular fat depot [38].

This study suggested that extracellular FA levels around brown adipocytes might be one of the systemic factors controlling UCP1 expression in human brown adipocytes. This regulation, at least partially, accounts for less active BAT in obese individuals than in lean individuals [3,4,14]. High blood-FA levels in obese individuals resemble continuous FA treatment. This study showed that FA supply was associated with the accumulation of intracellular triglycerides, high MMP, and the reduced expression of UCP1. In contrast, FA-depleted conditions resemble low blood-FA levels observed in lean individuals. The FA depletion was associated with reduced triglyceride content, low MMP, and the activated transcription of UCP1 and lipid metabolic genes, such as FADAGs. Collectively, the feedback regulation of UCP1 transcription may be beneficial for the maintenance of consistent heat production under various nutritional conditions. The insights obtained using human-brown-adipocyte models in this study are fundamental to properly evaluate and modulate the activity and function of human brown adipocytes. However, further studies are required for a deeper understanding of the biological significance of the UCP1 regulation in vivo.

Brown adipocyte is an attractive therapeutic target for the prevention of obesity and related metabolic diseases, including type 2 diabetes [39,40,41]. ciBAs present a promising cell model for the identification of bioactive molecules to promote adipocyte browning and brown adipogenesis in humans [31]. This study suggested the regulatory mechanism of UCP1 expression through cellular lipid metabolism, which may be indispensable to evaluate the browning effects during the screening of relevant molecules as anti-obesity drugs. However, elaborate experiments are required to determine the effects of candidate molecules on adipocyte browning and brown adipogenesis. Therefore, the regulatory mechanism underlying UCP1 expression may assist in identifying anti-obesity drugs as well as dietary compounds targeting human brown adipocytes.

## Data Availability

All RNA-sequencing data in this study have been deposited in the DNA Data Bank of Japan (DDBJ) Sequenced Read Archive (https://www.ddbj.nig.ac.jp/dra/index-e.html, last accessed on 20 May 2022) under the accession numbers DRA014166, DRA014311, and DRA014312.

## Supplementary Materials

The following are available online at https://www.mdpi.com/article/10.3390/cells11132038/s1. Figure S1: Characterization of ciBAs converted under FA-free conditions. Figure S2: Regulation of *UCP1* expression by treatment with various FAs and carnitine. Figure S3: Comparison of genome-wide transcriptional analysis in ciBAs converted by either FA BSA or FA-free BSA. Figure S4: Comparison of genome-wide transcriptional analysis in ciBAs treated with either carnitine or PA. Figure S5: RNA-Seq results on the expression of lipid metabolic genes and transcription factors. Figure S6: Characterization of adipocytes derived from hTERT A41BAT-SVF cells and AdMSC. Figure S7: Effects of oligomycin and FCCP on mitochondria-membrane potential (MMP) in ciBAs. Figure S8: Original images of full-length Western blots. Table S1: List of 137 fatty acid depletion-activated genes (FADAGs) in ciBAs. Table S2: Top 15 GO terms for 137 FADAGs. Table S3: Information on human dermal fibroblasts (HDFs) and mesenchymal stem cells (MSCs) used in this study. Table S4: Sequences and product size of primers used for qRT-PCR.

## References

[1] Cohen P., Kajimura S. (2021). The cellular and functional complexity of thermogenic fat. Nat. Rev. Mol. Cell Biol..

[2] Sidossis L., Kajimura S. (2015). Brown and beige fat in humans: Thermogenic adipocytes that control energy and glucose homeostasis. J. Clin. Investig..

[3] van Marken Lichtenbelt W.D., Vanhommerig J.W., Smulders N.M., Drossaerts J.M.A.F.L., Kemerink G.J., Bouvy N.D., Schrauwen P., Teule G.J.J. (2009). Cold-activated brown adipose tissue in healthy men. N. Engl. J. Med..

[4] Cypess A.M., Lehman S., Williams G., Tal I., Rodman D., Goldfine A.B., Kuo F.C., Palmer E.L., Tseng Y.H., Doria A. (2009). Identification and importance of brown adipose tissue in adult humans. N. Engl. J. Med..

[5] Saito M., Okamatsu-Ogura Y., Matsushita M., Watanabe K., Yoneshiro T., Nio-Kobayashi J., Iwanaga T., Miyagawa M., Kameya T., Nakada K. (2009). High incidence of metabolically active brown adipose tissue in healthy adult humans: Effects of cold exposure and adiposity. Diabetes.

[6] Oguri Y., Shinoda K., Kim H., Alba D.L., Bolus W.R., Wang Q., Brown Z., Pradhan R.N., Tajima K., Yoneshiro T. (2020). CD81 controls beige fat progenitor cell growth and energy balance via FAK signaling. Cell.

[7] Angueira A.R., Sakers A.P., Holman C.D., Cheng L., Arbocco M.N., Shamsi F., Lynes M.D., Shrestha R., Okada C., Batmanov K. (2020). Defining the lineage of thermogenic perivascular adipose tissue. Nat. Metab..

[8] Becher T., Palanisamy S., Kramer D.J., Eljalby M., Marx S.J., Wibmer A.G., Butler S.D., Jiang C.S., Vaughan R., Schöder H. (2021). Brown adipose tissue is associated with cardiometabolic health. Nat. Med..

[9] Orava J., Nuutila P., Lidell M.E., Oikonen V., Noponen T., Viljanen T., Scheinin M., Taittonen M., Niemi T., Enerbäck S. (2011). Different metabolic responses of human brown adipose tissue to activation by cold and insulin. Cell Metab..

[10] Ouellet V., Labbé S.M., Blondin D.P., Phoenix S., Guérin B., Haman F., Turcotte E.E., Richard D., Carpentier A.C. (2012). Brown adipose tissue oxidative metabolism contributes to energy expenditure during acute cold exposure in humans. J. Clin. Investig..

[11] Blondin D.P., Tingelstad H.C., Noll C., Frisch F., Phoenix S., Guérin B., Turcotte É.E., Richard D., Haman F., Carpentier A.C. (2017). Dietary fatty acid metabolism of brown adipose tissue in cold-acclimated men. Nat. Commun..

[12] Wibmer A.G., Becher T., Eljalby M., Crane A., Andrieu P.C., Jiang C.S., Vaughan R., Schöder H., Cohen P. (2021). Brown adipose tissue is associated with healthier body fat distribution and metabolic benefits independent of regional adiposity. Cell Rep. Med..

[13] Ahmed B.A., Ong F.J., Barra N.G., Blondin D.P., Gunn E., Oreskovich S.M., Szamosi J.C., Syed S.A., Hutchings E.K., Konyer N.B. (2021). Lower brown adipose tissue activity is associated with non-alcoholic fatty liver disease but not changes in the gut microbiota. Cell Rep. Med..

[14] Leitner B.P., Huang S., Brychta R.J., Duckworth C.J., Baskin A.S., McGehee S., Tal I., Dieckmann W., Gupta G., Kolodny G.M. (2017). Mapping of human brown adipose tissue in lean and obese young men. Proc. Natl. Acad. Sci. USA.

[15] Orava J., Nuutila P., Noponen T., Parkkola R., Viljanen T., Enerbäck S., Rissanen A., Pietiläinen K.H., Virtanen K.A. (2013). Blunted metabolic responses to cold and insulin stimulation in brown adipose tissue of obese humans. Obesity (Silver Spring).

[16] Din M.U., Raiko J., Saari T., Saunavaara V., Kudomi N., Solin O., Parkkola R., Nuutila P., Virtanen K.A. (2017). Human brown fat radiodensity indicates underlying tissue composition and systemic metabolic health. J. Clin. Endocrinol. Metab..

[17] Saari T.J., Raiko J., U-Din M., Niemi T., Taittonen M., Laine J., Savisto N., Haaparanta-Solin M., Nuutila P., Virtanen K.A. (2020). Basal and cold-induced fatty acid uptake of human brown adipose tissue is impaired in obesity. Sci. Rep..

[18] Mengel L.A., Nemati Moud B., Seidl H., Mesas-Fernández A., Seeliger C., Brandl B., Skurk T., Holzapfel C., Claussnitzer M., Hauner H. (2022). Effect of BMI on the thermogenic response to cold exposure and associated changes in metabolism and browning markers in adult humans. Obes. Facts.

[19] Takahashi A., Adachi S., Morita M., Tokumasu M., Natsume T., Suzuki T., Yamamoto T. (2015). Post-transcriptional stabilization of Ucp1 mRNA protects mice from diet-induced obesity. Cell Rep..

[20] Fromme T., Klingenspor M. (2011). Uncoupling protein 1 expression and high-fat diets. Am. J. Physiol-Regul. Integr. Comp. Physiol..

[21] Li L., Ma L., Zhao Z., Luo S., Gong B., Li J., Feng J., Zhang H., Qi W., Zhou T. (2021). IL-25-induced shifts in macrophage polarization promote development of beige fat and improve metabolic homeostasis in mice. PLoS Biol..

[22] Wang Q., Li D., Cao G., Shi Q., Zhu J., Zhang M., Cheng H., Wen Q., Xu H., Zhu L. (2021). IL-27 signalling promotes adipocyte thermogenesis and energy expenditure. Nature.

[23] Wankhade U.D., Lee J.H., Dagur P.K., Yadav H., Shen M., Chen W., Kulkarni A.B., McCoy J.P., Finkel T., Cypess A.M. (2018). TGF-β receptor 1 regulates progenitors that promote browning of white fat. Mol. Metab..

[24] Bi P., Shan T., Liu W., Yue F., Yang X., Liang X.R., Wang J., Li J., Carlesso N., Liu X. (2014). Inhibition of Notch signaling promotes browning of white adipose tissue and ameliorates obesity. Nat. Med..

[25] Oo S.M., Oo H.K., Takayama H., Ishii K.A., Takeshita Y., Goto H., Nakano Y., Kohno S., Takahashi C., Nakamura H. (2022). Selenoprotein P-mediated reductive stress impairs cold-induced thermogenesis in brown fat. Cell Rep..

[26] Samuelson I., Vidal-Puig A. (2020). Studying brown adipose tissue in a human in vitro context. Front. Endocrinol. (Lausanne).

[27] Takeda Y., Harada Y., Yoshikawa T., Dai P. (2017). Direct conversion of human fibroblasts to brown adipocytes by small chemical compounds. Sci. Rep..

[28] Takeda Y., Dai P. (2020). A developed serum-free medium and an optimized chemical cocktail for direct conversion of human dermal fibroblasts into brown adipocytes. Sci. Rep..

[29] Blondin D.P., Frisch F., Phoenix S., Guérin B., Turcotte É.E., Haman F., Richard D., Carpentier A.C. (2017). Inhibition of intracellular triglyceride lipolysis suppresses cold-induced brown adipose tissue metabolism and increases shivering in humans. Cell Metab..

[30] Schneider C.A., Rasband W.S., Eliceiri K.W. (2012). NIH Image to ImageJ: 25 years of image analysis. Nat. Methods.

[31] Babicki S., Arndt D., Marcu A., Liang Y., Grant J.R., Maciejewski A., Wishart D.S. (2016). Heatmapper: Web-enabled heat mapping for all. Nucleic Acids Res..

[32] Huang D.W., Sherman B.T., Lempicki R.A. (2009). Systematic and integrative analysis of large gene lists using DAVID bioinformatics resources. Nat. Protoc..

[33] Takeda Y., Yoshikawa T., Dai P. (2021). Transcriptome analysis reveals brown adipogenic reprogramming in chemical compound-induced brown adipocytes converted from human dermal fibroblasts. Sci. Rep..

[34] Takeda Y., Dai P. (2022). Capsaicin directly promotes adipocyte browning in the chemical compound-induced brown adipocytes converted from human dermal fibroblasts. Sci. Rep..

[35] Verkerke A.R.P., Kajimura S. (2021). Oil does more than light the lamp: The multifaceted role of lipids in thermogenic fat. Dev. Cell.

[36] Yang H., van der Stel W., Lee R., Bauch C., Bevan S., Walker P., van de Water B., Danen E.H.J., Beltman J.B. (2021). Dynamic modeling of mitochondrial membrane potential upon exposure to mitochondrial inhibitors. Front. Pharmacol..

[37] Zulian A., Tagliavini F., Rizzo E., Pellegrini C., Sardone F., Zini N., Maraldi N.M., Santi S., Faldini C., Merlini L. (2014). Melanocytes from patients affected by Ullrich congenital muscular dystrophy and Bethlem myopathy have dysfunctional mitochondria that can be rescued with cyclophilin inhibitors. Front. Aging Neurosci..

[38] Dadson P., Hannukainen J.C., Din M.U., Lahesmaa M., Kalliokoski K.K., Iozzo P., Pihlajamäki J., Karlsson H.K., Parkkola R., Salminen P. (2018). Brown adipose tissue lipid metabolism in morbid obesity: Effect of bariatric surgery-induced weight loss. Diabetes Obes. Metab..

[39] Singh A.M., Zhang L., Avery J., Yin A., Du Y., Wang H., Li Z., Fu H., Yin H., Dalton S. (2020). Human beige adipocytes for drug discovery and cell therapy in metabolic diseases. Nat. Commun..

[40] Singh R., Barrios A., Dirakvand G., Pervin S. (2021). Human brown adipose tissue and metabolic health: Potential for therapeutic avenues. Cells.

[41] Wang B., Tsakiridis E.E., Zhang S., Llanos A., Desjardins E.M., Yabut J.M., Green A.E., Day E.A., Smith B.K., Lally J.S.V. (2021). The pesticide chlorpyrifos promotes obesity by inhibiting diet-induced thermogenesis in brown adipose tissue. Nat. Commun..

