# Supplementary material for: Chronic Fatty Acid Depletion Induces Uncoupling Protein 1 (UCP1) Expression to Coordinate Mitochondrial Inducible Proton Leak in a Human-Brown-Adipocyte Model
Source: Cells. 2022 Jun 27;11(13):2038. doi: 10.3390/cells11132038 (PMC9265531; doi:10.3390/cells11132038)
Supplement: Supplementary file 1 [file cells-11-02038-s001.zip › cells-1779269-supplementary.pdf]

Supplementary Materials

Chronic Fatty Acid Depletion Induces Uncoupling Protein 1 (UCP1) Expression to Coordinate Mitochondrial Inducible Proton Leak in a Human-Brown-Adipocyte Model

Yukimasa Takeda and Ping Dai

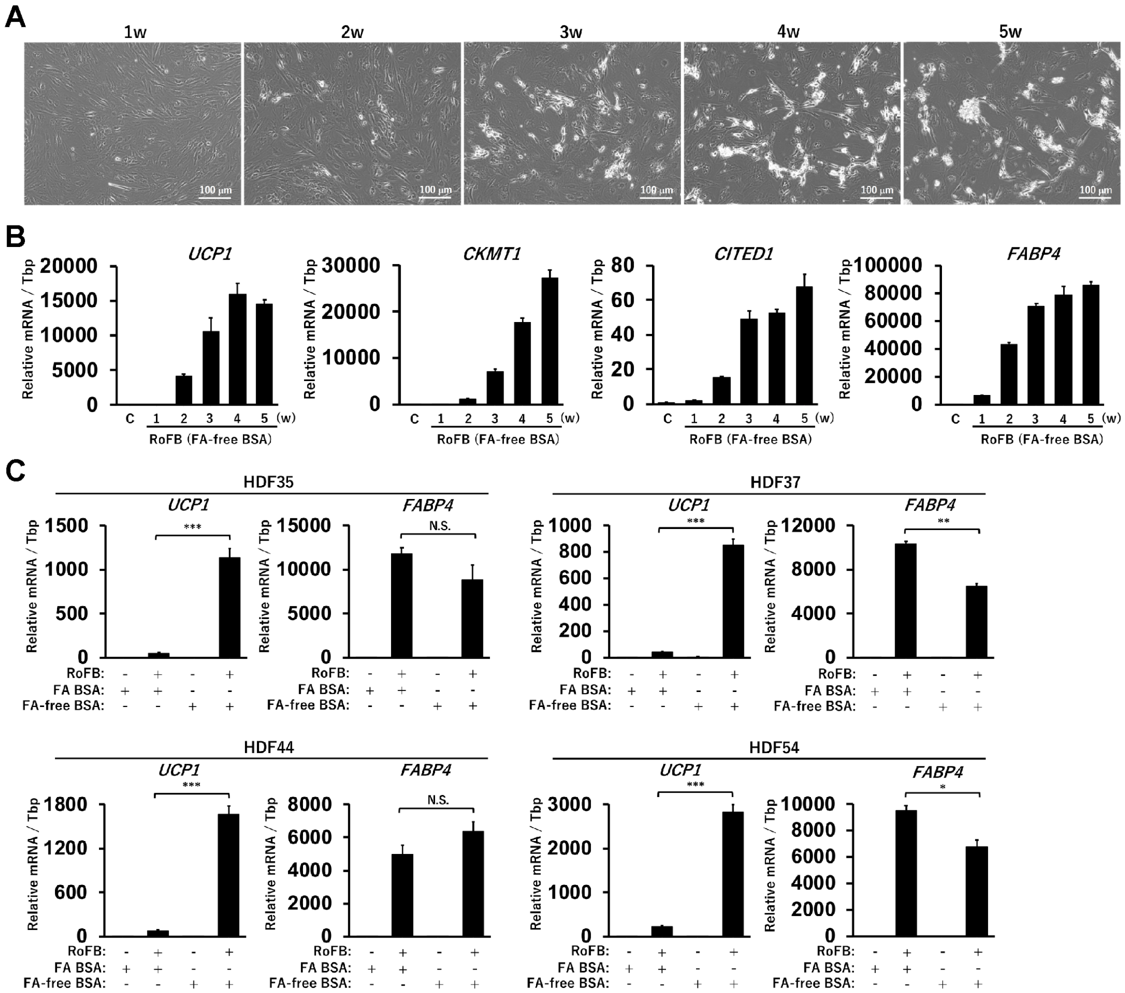

Figure S1. Characterization of ciBAs converted under FA-free conditions. (**A**) Representative pictures of ciBAs converted by RoFB and FA-free BSA for up to 5 weeks. (**B**) The time course of the expression of *UCP1*, *CKMT1*, *CITED1*, and *FABP4* was measured using qRT-PCR. "C" indicates the control fibroblasts cultured by the medium only. (**C**) The four lines of human dermal fibroblasts (HDFs) derived from different subjects aged 35, 37, 44, and 54 were converted into ciBAs by either FA-binding BSA or FA-free BSA for 3 weeks. The expression of *UCP1* and *FABP4* was quantitated using qRT-PCR. Data represent mean ± SD (n = 3). Student's t-test: *p < 0.05, **p < 0.01, ***p < 0.001, N.S.; not significant.

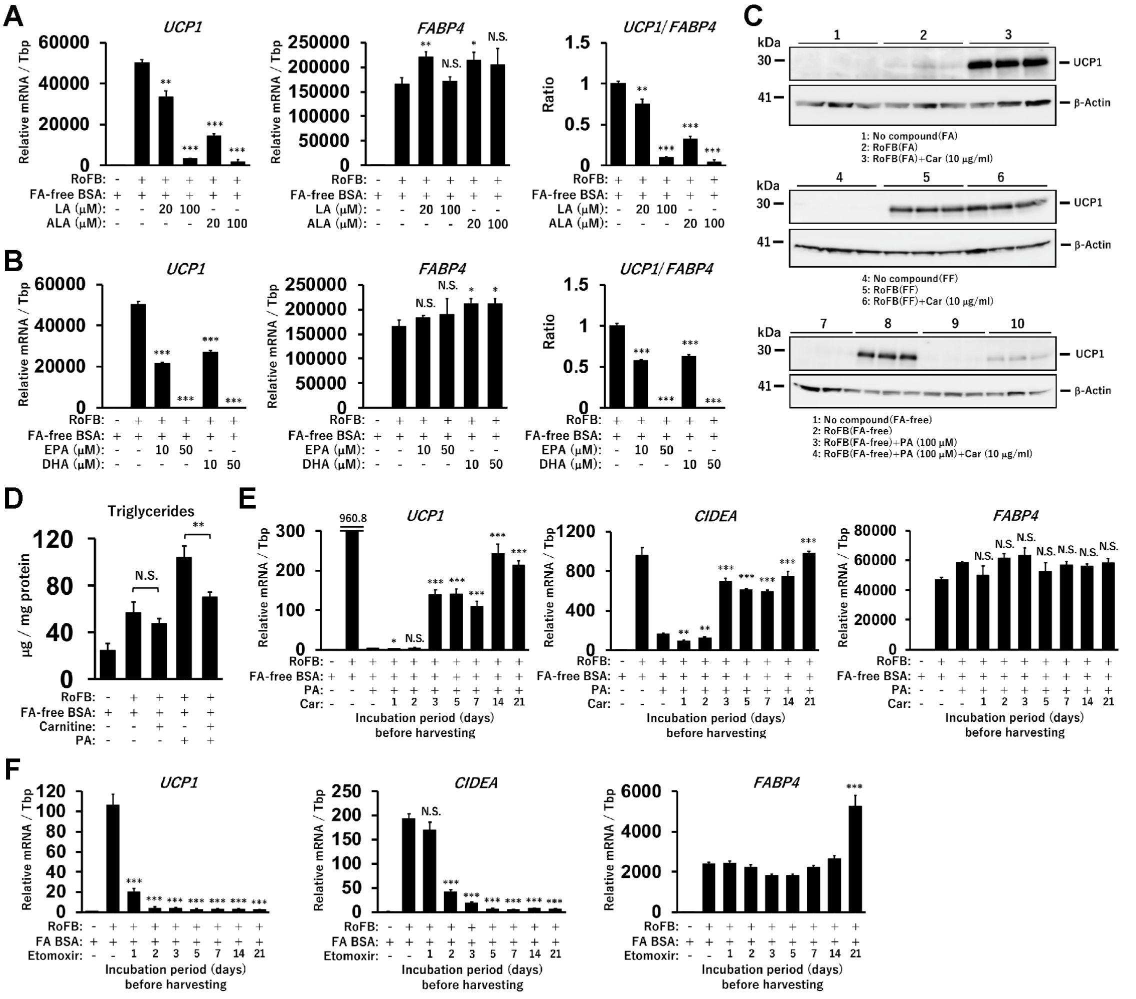

Figure S2. Regulation of *UCP1* expression by the treatment with various FAs and carnitine. (**A**) The expression of *UCP1* and *FABP4* mRNA and the ratio were quantified using qRT-PCR in ciBAs treated with either linoleic acid (LA) or α-linolenic acid (ALA) (20 and 100 μM). (**B**) The expression was quantified in ciBAs treated with either eicosapentaenoic acid (EPA) or docosahexaenoic acid (DHA) (10 and 50 μM). (**C**) The UCP1 and β-Actin proteins were immunoblotted in ciBAs treated with carnitine or PA. The quantification of the band intensities is shown in Figure 2F by densitometry using ImageJ software. (**D**) Cellular triglyceride content was measured in ciBAs converted by FA-free BSA under the treatment with carnitine and PA. (**E**) The expression of *UCP1*, *CIDEA*, and *FABP4* was measured using qRT-PCR in ciBAs treated with FA-free BSA, PA (100 μM), and carnitine (10 μg/ml) for different incubation periods (days) before harvesting the cells on day 21. (**F**) The expression was measured in ciBAs treated with

FA-binding BSA and etomoxir (50 μM) for different incubation periods (days) before harvesting the cells on day 21. Data represent mean ± SD (n = 3). Student's t-test: $*p < 0.05$, $**p < 0.01$, $***p < 0.001$, N.S.; not significant.

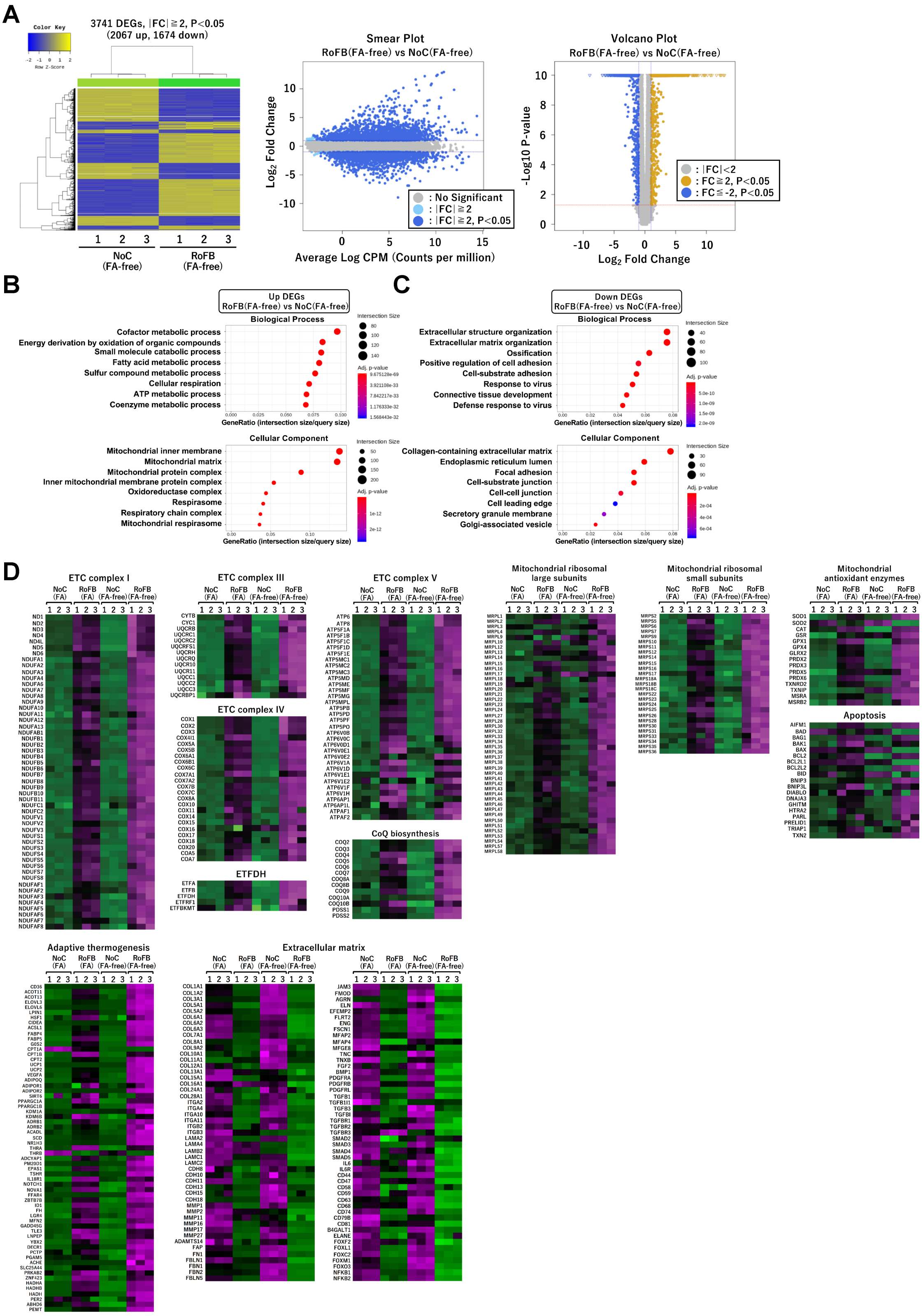

A
Color Key
Row Z-Score
3741 DEGs, |FC|≧2, P<0.05
(2067 up, 1674 down)
1 2 3
NoC
(FA-free)
RoFB
(FA-free)
Smear Plot
RoFB(FA-free) vs NoC(FA-free)
Log2 Fold Change
Average Log CPM (Counts per million)
: No Significant
: |FC|≧2
: |FC|≧2, P<0.05
Volcano Plot
RoFB(FA-free) vs NoC(FA-free)
-Log10 P-value
Log2 Fold Change
: |FC|<2
: FC≧2, P<0.05
: FC≦-2, P<0.05
B
Up DEGs
RoFB(FA-free) vs NoC(FA-free)
Biological Process
Cofactor metabolic process
Energy derivation by oxidation of organic compounds
Small molecule catabolic process
Fatty acid metabolic process
Sulfur compound metabolic process
Cellular respiration
ATP metabolic process
Coenzyme metabolic process
GeneRatio (intersection size/query size)
Intersection Size
Adj. p-value
Cellular Component
Mitochondrial inner membrane
Mitochondrial matrix
Mitochondrial protein complex
Inner mitochondrial membrane protein complex
Oxidoreductase complex
Respirasome
Respiratory chain complex
Mitochondrial respirasome
C
Down DEGs
RoFB(FA-free) vs NoC(FA-free)
Biological Process
Extracellular structure organization
Extracellular matrix organization
Ossification
Positive regulation of cell adhesion
Cell-substrate adhesion
Response to virus
Connective tissue development
Defense response to virus
Cellular Component
Collagen-containing extracellular matrix
Endoplasmic reticulum lumen
Focal adhesion
Cell-substrate junction
Cell-cell junction
Cell leading edge
Secretory granule membrane
Golgi-associated vesicle
D
ETC complex I
ETC complex III
ETC complex IV
ETFDH
ETC complex V
CoQ biosynthesis
Mitochondrial ribosomal large subunits
Mitochondrial ribosomal small subunits
Mitochondrial antioxidant enzymes
Apoptosis
Adaptive thermogenesis
Extracellular matrix
NoC (FA)
RoFB (FA)
NoC (FA-free)
RoFB (FA-free)

Figure S3. Comparison of genome-wide transcriptional analysis in ciBAs converted by either FA BSA or FA-free BSA. (**A**) Heatmap and hierarchical clustering analysis represent 3741 differentially expressed genes (DEGs) (|fold change (FC)| ≥ 2, $p < 0.05$) between RoFB(FA-free) ciBAs and NoC(FA-free) control fibroblasts. Smear and Volcano plots indicate logarithmic fold change, *P*-value, and counts per million (CPM) between RoFB(FA-free) and NoC(FA-free). (**B**,**C**) Gene ontology (GO) enrichment analysis was performed in 2067 upregulated DEGs (**B**) and 1674 downregulated DEGs (**C**). The top 10 GO terms are represented in the category of biological process and cellular component. (**D**) Heatmaps represent transcriptional profiles in functional groups related to the electron transfer chain (ETC) complex, mitochondrial ribosomal subunits, antioxidant enzymes, apoptosis, adaptive thermogenesis, and extracellular matrix in NoC(FA), RoFB(FA), NoC(FA-free), and RoFB(FA-free). The color scale shows z-scored fragments per kilobase of transcript per million mapped sequence reads (FPKM) representing mRNA levels of each gene in green (lower expression) and magenta (higher expression).

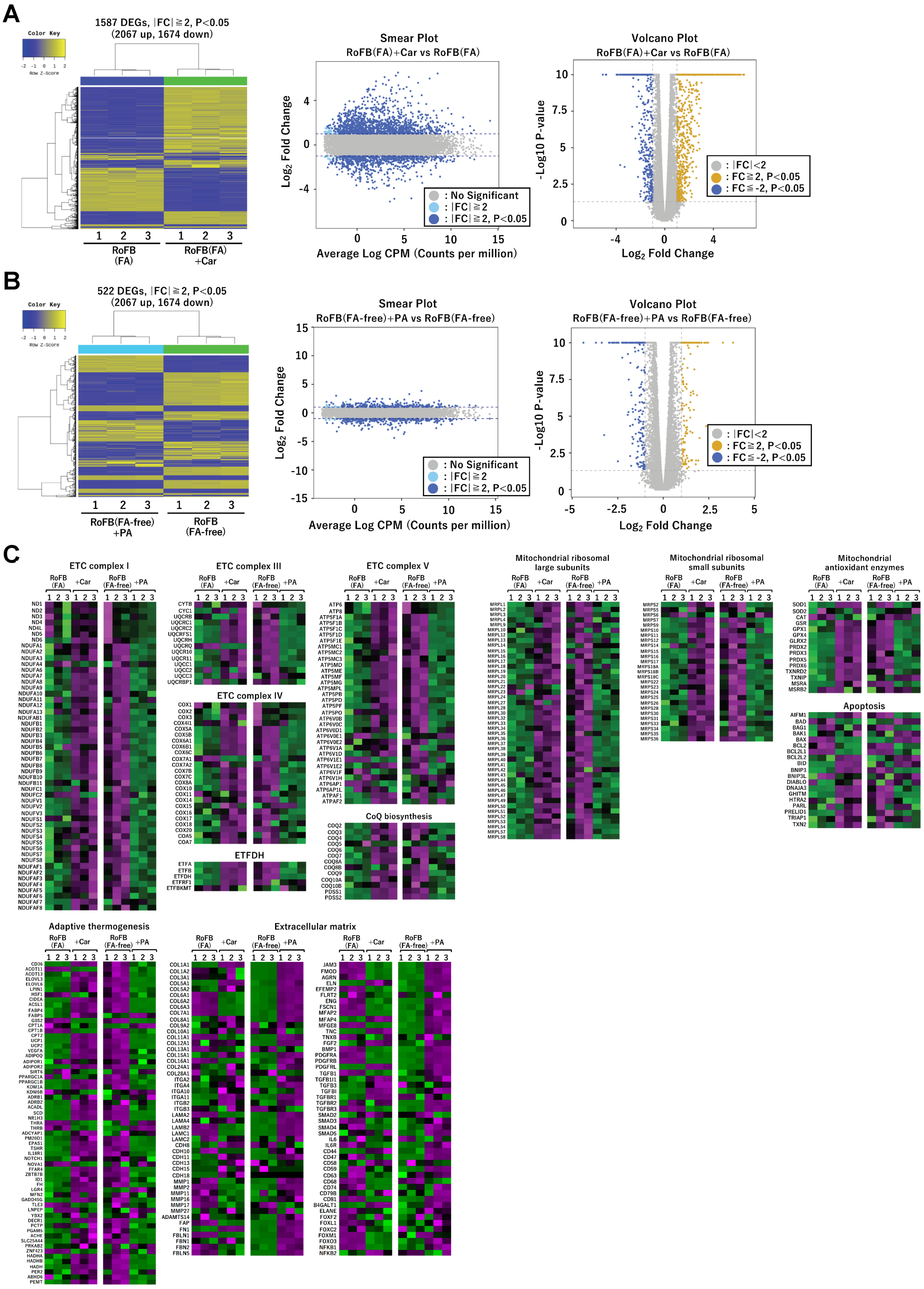
A
1587 DEGs, |FC|≧2, P<0.05
(2067 up, 1674 down)
Color Key
Row Z-Score
1 2 3 1 2 3
RoFB (FA)
RoFB(FA) +Car
Smear Plot
RoFB(FA)+Car vs RoFB(FA)
Log2 Fold Change
Average Log CPM (Counts per million)
: No Significant
: |FC|≧2
: |FC|≧2, P<0.05
Volcano Plot
RoFB(FA)+Car vs RoFB(FA)
-Log10 P-value
Log2 Fold Change
: |FC|<2
: FC≧2, P<0.05
: FC≦-2, P<0.05
B
522 DEGs, |FC|≧2, P<0.05
(2067 up, 1674 down)
Color Key
Row Z-Score
RoFB(FA-free) +PA
RoFB (FA-free)
Smear Plot
RoFB(FA-free)+PA vs RoFB(FA-free)
Log2 Fold Change
Average Log CPM (Counts per million)
: No Significant
: |FC|≧2
: |FC|≧2, P<0.05
Volcano Plot
RoFB(FA-free)+PA vs RoFB(FA-free)
-Log10 P-value
Log2 Fold Change
: |FC|<2
: FC≧2, P<0.05
: FC≦-2, P<0.05
C
ETC complex I
ETC complex III
ETC complex IV
ETFDH
ETC complex V
CoQ biosynthesis
Mitochondrial ribosomal large subunits
Mitochondrial ribosomal small subunits
Mitochondrial antioxidant enzymes
Apoptosis
Adaptive thermogenesis
Extracellular matrix
RoFB (FA)
+Car
RoFB (FA-free)
+PA

Figure S4. Comparison of genome-wide transcriptional analysis in ciBAs treated with either carnitine or PA. (**A**) Heatmap and hierarchical clustering analysis represent 1587 DEGs ($|FC| \geq 2$, $p < 0.05$) between RoFB(FA) and RoFB(FA)+Car. Smear and Volcano plots indicate logarithmic FC, P-value, and CPM between them. (**B**) 522 DEGs were detected between RoFB(FA-free) and RoFB(FA-free)+PA. (**C**) Heatmaps represent transcriptional profiles in the functional groups, as indicated, between RoFB(FA) and RoFB(FA)+Car and between RoFB(FA-free) and RoFB(FA-free)+PA. The color scale shows z-scored FPKM representing mRNA levels of each gene in green (lower expression) and magenta (higher expression).

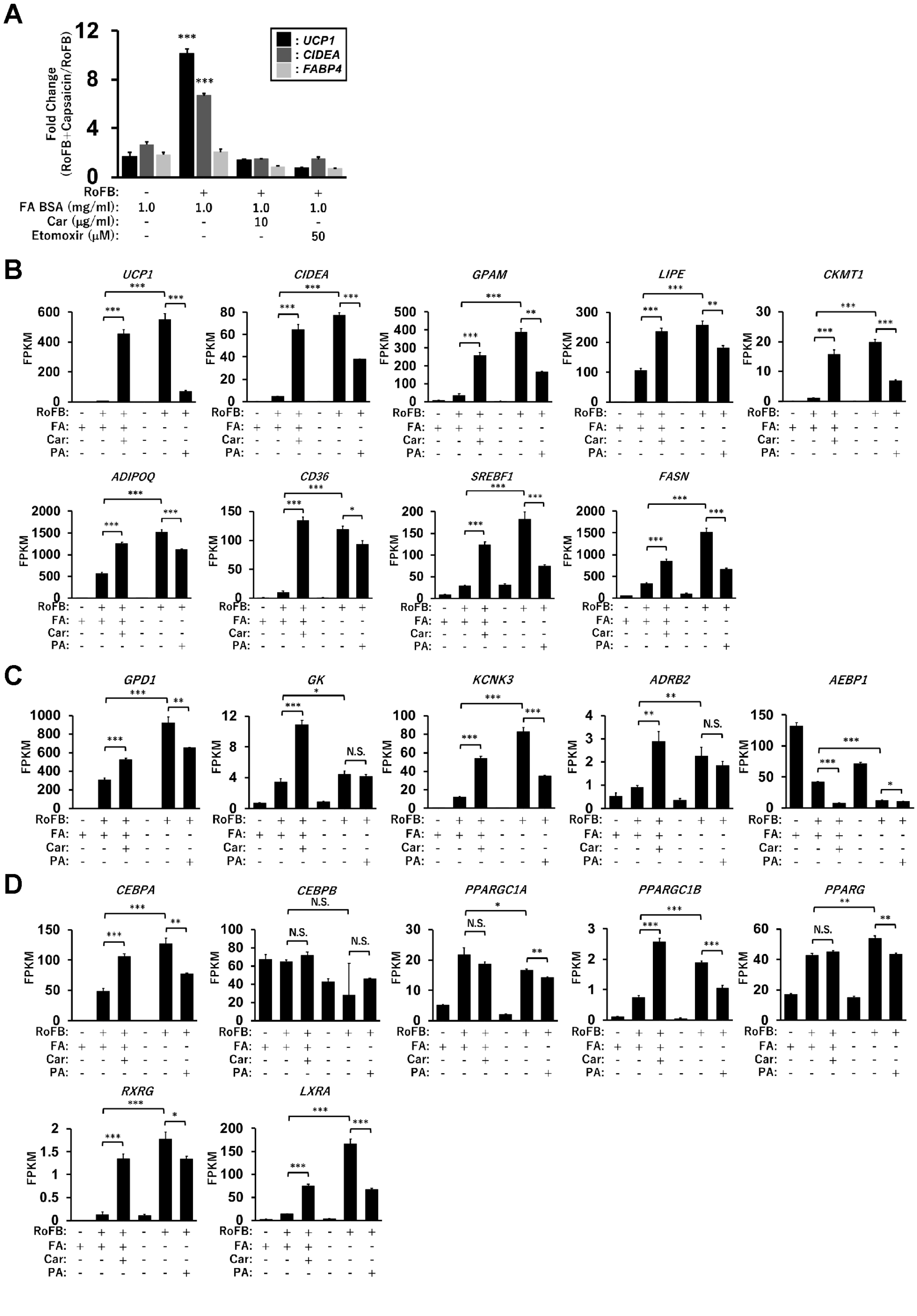

Figure S5. RNA-Seq results on the expression of lipid metabolic genes and transcription factors. (**A**) The fold change of *UCP1*, *CIDEA*, and *FABP4* expression by capsaicin was quantified in ciBAs treated with either carnitine (10 μg/ml) or etomoxir

(50 μM). (**B,C**) The FPKM values of *UCP1*, *CIDEA*, *GPAM*, *LIPE*, *CKMT1*, *ADIPOQ*, *CD36*, *SREBF1*, and *FASN* (**B**), as well as *GPD1*, *GK*, *KCNK3*, *ADBR2*, and *AEBP1* (**C**) were depicted from the RNA-Seq results to compare corresponding qRT-PCR results in Figures 4E and 4F. (**D**) The FPKM values of *CEBPA*, *CEBPB*, *PPARGC1A*, *PPARGC1B*, *PPARG*, *RXRG*, and *LXRA* were also depicted to compare corresponding qRT-PCR results in Figure 4G. Data represent mean ± SD ($n = 3$). Student's t-test: *$p < 0.05$, **$p < 0.01$, ***$p < 0.001$, N.S.; not significant.

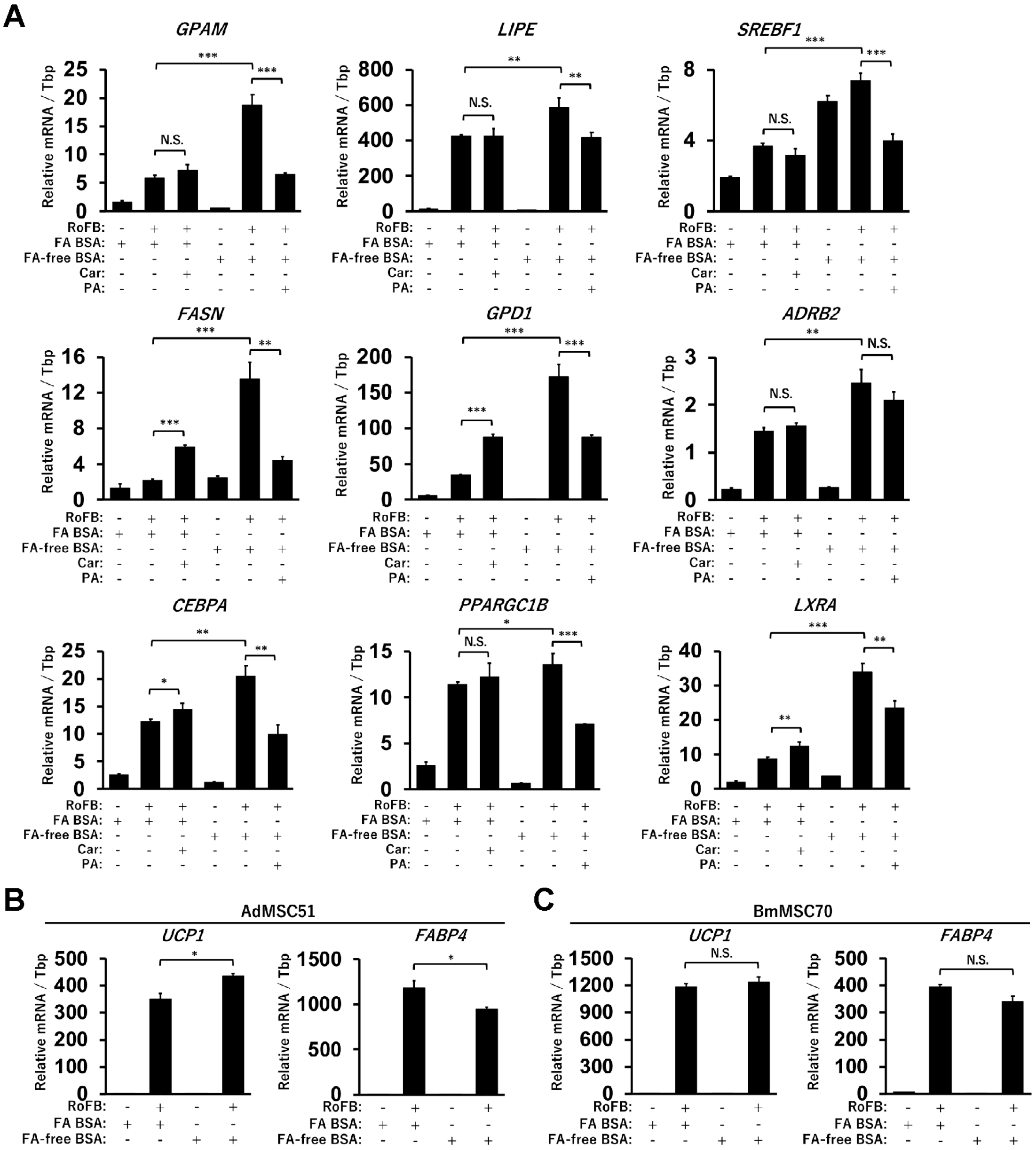

Figure S6. Characterization of adipocytes derived from hTERT A41BAT-SVF cells and AdMSC. (**A**) The expression of *GPAM*, *LIPE*, *SREBF1*, *FASN*, *GPD1*, *ADBR2*, *CEBPA*, *PPARGC1B*, and *LXRA* was quantified using qRT-PCR in A41BAT-SVF adipocytes differentiated under various conditions, as indicated. (**B,C**) The expression of *UCP1* and *FABP4* was quantified in mature adipocytes differentiated from human MSCs derived from adipose tissue (AdMSC51) (**B**) and bone marrow (BmMSC70) (**C**). The information on the MSCs is listed in Table S3. Data represent mean ± SD (n = 3). Student's t-test: *$p < 0.05$, **$p < 0.01$, ***$p < 0.001$, N.S.; not significant.

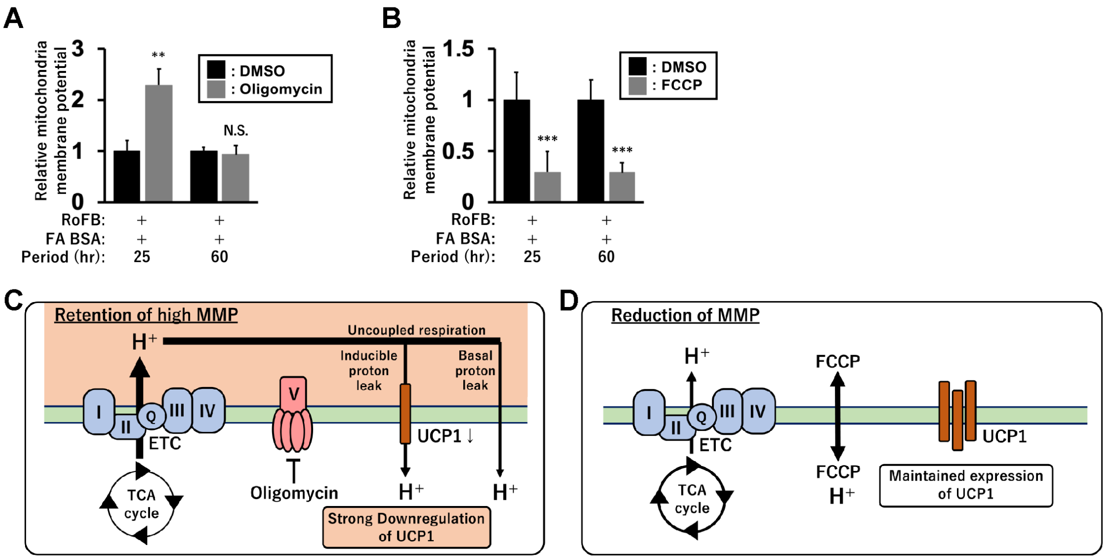

Figure S7. Effects of oligomycin and FCCP on mitochondria membrane potential (MMP) in ciBAs. (**A**) Relative MMP levels were measured in ciBAs, 1 and 36 h after the treatment with either DMSO or oligomycin. The period (hr) follows the illustration in Figure 7E. The area of staining of MT-1 dye was quantified using ImageJ software. (**B**) Relative MMP levels were similarly measured in ciBAs 1 and 36 hours after the treatment with either DMSO or FCCP. (**C,D**) Schematic presentations represent a possible status of mitochondrial inner membrane in the treatment with oligomycin (**C**) or FCCP (**D**). Oligomycin leads to the retention of MMP at a high level by inhibiting the dissipation of a proton gradient. In this condition, *UCP1* expression was strongly downregulated likely to avoid an excess amount of inducible proton leak by UCP1. The uncoupler FCCP reduces MMP by releasing a proton gradient across mitochondrial inner membrane. In this condition, *UCP1* expression was maintained.

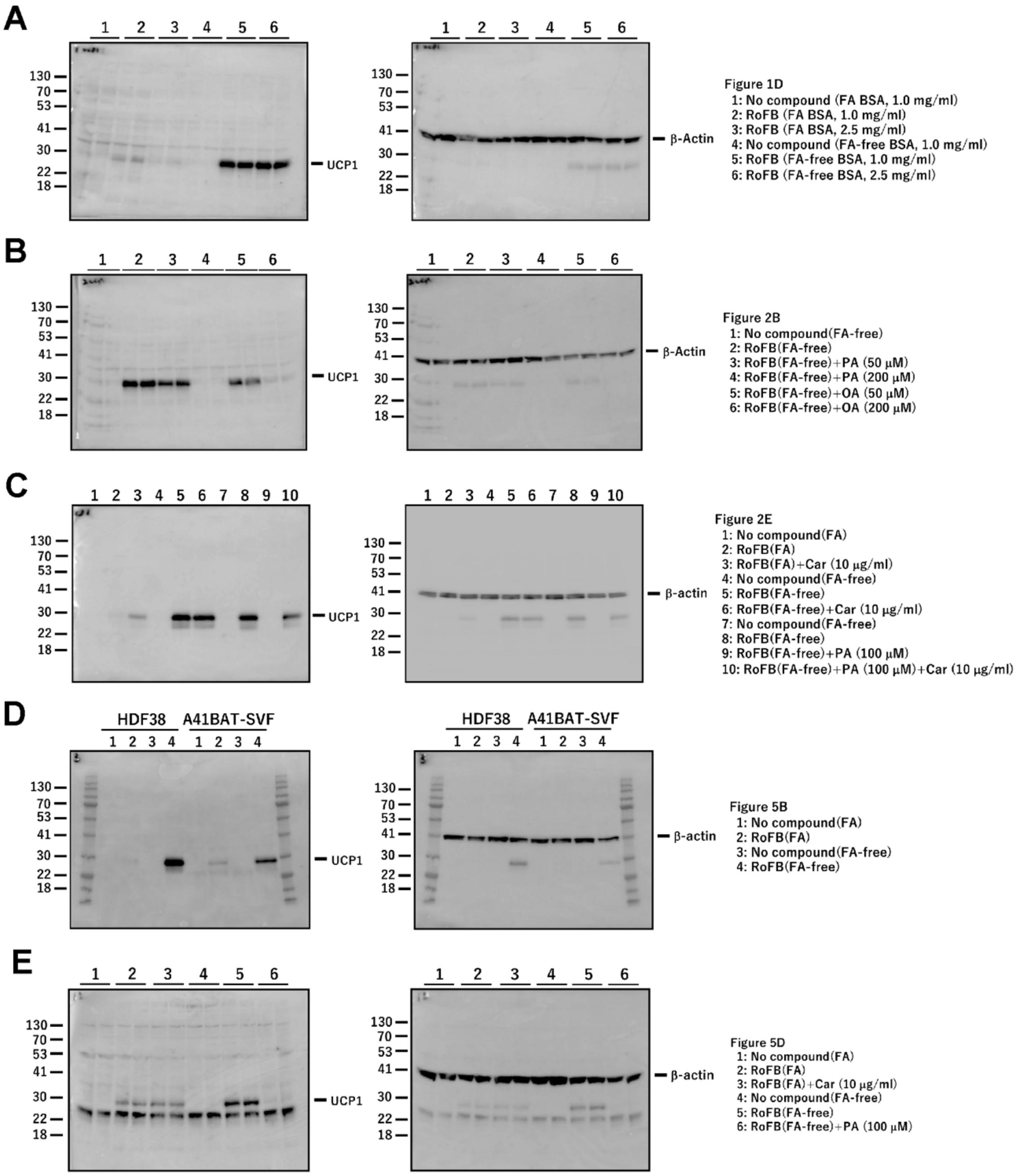
A
UCP1
β-Actin
Figure 1D
1: No compound (FA BSA, 1.0 mg/ml)
2: RoFB (FA BSA, 1.0 mg/ml)
3: RoFB (FA BSA, 2.5 mg/ml)
4: No compound (FA-free BSA, 1.0 mg/ml)
5: RoFB (FA-free BSA, 1.0 mg/ml)
6: RoFB (FA-free BSA, 2.5 mg/ml)
B
UCP1
β-Actin
Figure 2B
1: No compound(FA-free)
2: RoFB(FA-free)
3: RoFB(FA-free)+PA (50 μM)
4: RoFB(FA-free)+PA (200 μM)
5: RoFB(FA-free)+OA (50 μM)
6: RoFB(FA-free)+OA (200 μM)
C
UCP1
β-actin
Figure 2E
1: No compound(FA)
2: RoFB(FA)
3: RoFB(FA)+Car (10 μg/ml)
4: No compound(FA-free)
5: RoFB(FA-free)
6: RoFB(FA-free)+Car (10 μg/ml)
7: No compound(FA-free)
8: RoFB(FA-free)
9: RoFB(FA-free)+PA (100 μM)
10: RoFB(FA-free)+PA (100 μM)+Car (10 μg/ml)
D
HDF38
A41BAT-SVF
UCP1
β-actin
Figure 5B
1: No compound(FA)
2: RoFB(FA)
3: No compound(FA-free)
4: RoFB(FA-free)
E
UCP1
β-actin
Figure 5D
1: No compound(FA)
2: RoFB(FA)
3: RoFB(FA)+Car (10 μg/ml)
4: No compound(FA-free)
5: RoFB(FA-free)
6: RoFB(FA-free)+PA (100 μM)

F

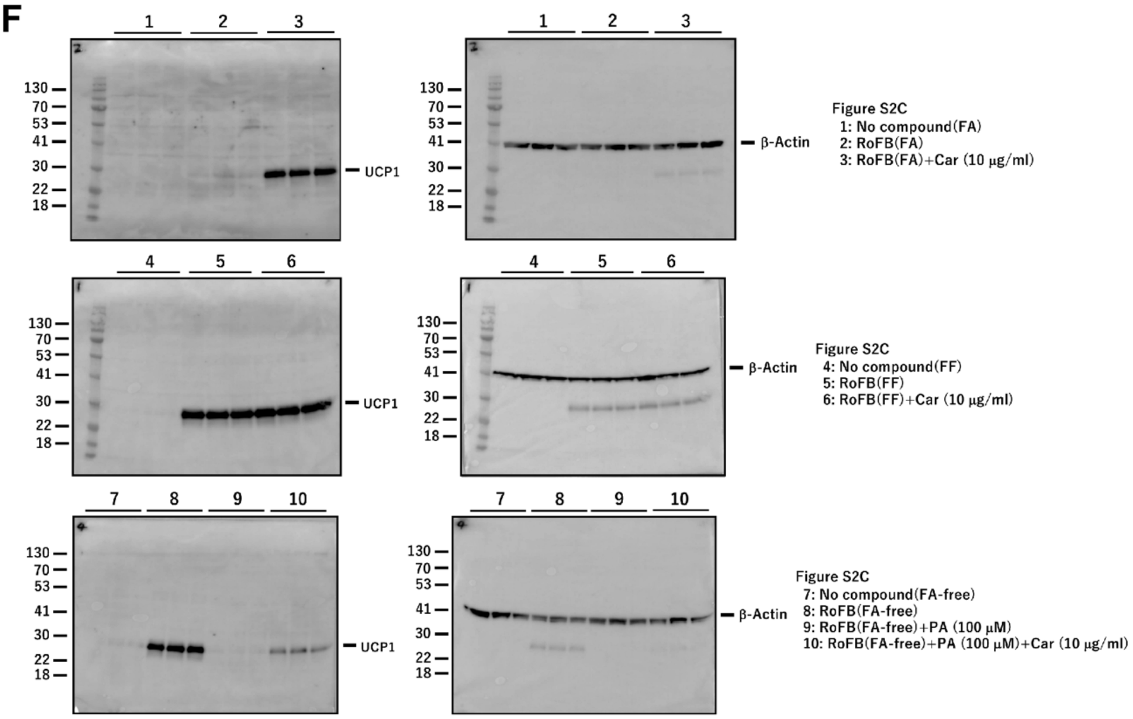

Figure S8. Original images of full-length western blots. They are shown for Figure 1D (**A**), Figure 2B (**B**), Figure 2E (**C**), Figure 5B (**D**), Figure 5D (**E**), and Figure S2C (**F**).

Table S1. List of 137 fatty acid depletion-activated genes (FADAGs) in ciBAs. The fold changes were calculated from the expression levels between RoFB(FA) and RoFB(FA-free).

Gene name	Description	Fold Change	Adjusted p-value
SLC2A5	Solute carrier family 2 member 5	143.58	0.00E+00
TF	Transferrin	111.23	8.16E-240
PDZD2	PDZ domain containing 2	91.76	0.00E+00
SHISA9	Shisa family member 9	58.31	6.67E-243
THRSP	Thyroid hormone responsive	24.93	0.00E+00
PLA2G2A	Phospholipase A2 group IIA	22.94	7.12E-193
ME1	Malic enzyme 1	20.83	0.00E+00
ALDH5A1	Aldehyde dehydrogenase 5 family member A1	19.87	7.64E-220
MARCO	Macrophage receptor with collagenous structure	17.78	1.25E-224
APOC1	Apolipoprotein C1	17.42	1.09E-242
TMEM37	Transmembrane protein 37	17.10	1.19E-227
CACNA2D4	Calcium voltage-gated channel auxiliary subunit alpha2delta 4	16.37	3.28E-68
SORL1	Sortilin related receptor 1	14.64	9.29E-152
ACSS2	Acyl-CoA synthetase short chain family member 2	14.27	0.00E+00
PRRT4	Proline rich transmembrane protein 4	14.10	3.06E-244
LBHD1	LBH domain containing 1	13.77	3.82E-69
NR1H3	Nuclear receptor subfamily 1 group H member 3	13.72	1.00E-280
ALDH1L1	Aldehyde dehydrogenase 1 family member L1	13.71	0.00E+00
ETNK2	Ethanolamine kinase 2	13.58	2.09E-249
GLYCTK	Glycerate kinase	13.50	4.31E-45
GPAM	Glycerol-3-phosphate acyltransferase, mitochondrial	11.99	5.86E-190
SULT1B1	Sulfotransferase family 1B member 1	11.87	1.76E-39
CLMN	Calmin	11.71	0.00E+00
ACLY	ATP citrate lyase	11.70	0.00E+00
SCARB1	Scavenger receptor class B member 1	11.44	9.37E-125
ABCC2	ATP binding cassette subfamily C member 2	11.31	2.59E-180
RIMS4	Regulating synaptic membrane exocytosis 4	10.94	1.34E-67
DHRS9	Dehydrogenase/reductase 9	10.84	3.99E-103
TSHR	Thyroid stimulating hormone receptor	10.82	2.41E-210
SLC29A4	Solute carrier family 29 member 4	10.40	1.90E-248
CD14	CD14 molecule	9.51	6.85E-98
PNPLA3	Patatin like phospholipase domain containing 3	9.32	3.56E-221
RBP4	Retinol binding protein 4	9.22	3.00E-192
LOC100130872	Uncharacterized LOC100130872	9.04	1.56E-40

FADS2	Fatty acid desaturase 2	8.31	1.05E-237
ACSL5	Acyl-CoA synthetase long chain family member 5	8.27	4.10E-131
GFOD1	Glucose-fructose oxidoreductase domain containing 1	7.81	2.13E-131
SYN2	Synapsin II	7.70	8.74E-216
KCNK3	Potassium two pore domain channel subfamily K member 3	7.55	2.72E-297
HCP5	HLA complex P5	7.50	2.42E-23
STBD1	Starch binding domain 1	7.49	1.09E-05
IQGAP3	IQ motif containing GTPase activating protein 3	7.44	1.03E-34
SCD	Stearoyl-CoA desaturase	7.25	1.67E-217
OASL	2'-5'-oligoadenylate synthetase like	7.06	1.86E-105
SREBF1	Sterol regulatory element binding transcription factor 1	6.99	8.96E-171
ARRB1	Arrestin beta 1	6.92	3.27E-232
FAR2	Fatty acyl-CoA reductase 2	6.88	5.15E-240
COL5A3	Collagen type V alpha 3 chain	6.86	1.25E-290
SCCPDH	Saccharopine dehydrogenase (putative)	6.81	2.05E-301
INSIG1	Insulin induced gene 1	6.75	3.04E-213
NES	Nestin	6.67	8.35E-24
LPCAT3	Lysophosphatidylcholine acyltransferase 3	6.45	3.91E-252
PDK1	Pyruvate dehydrogenase kinase 1	6.40	9.15E-81
TSPAN1	Tetraspanin 1	6.35	3.46E-23
FGR	FGR proto-oncogene, Src family tyrosine kinase	6.30	1.37E-159
MAFF	MAF bZIP transcription factor F	6.19	1.54E-147
ELOVL3	ELOVL fatty acid elongase 3	6.00	8.77E-145
TM7SF2	Transmembrane 7 superfamily member 2	5.95	2.79E-154
PTK2B	Protein tyrosine kinase 2 beta	5.87	8.61E-234
LPL	Lipoprotein lipase	5.79	3.47E-230
SLC4A4	Solute carrier family 4 member 4	5.78	5.25E-117
AATBC	Apoptosis associated transcript in bladder cancer	5.78	3.21E-21
NXPH4	Neurexophilin 4	5.78	3.59E-21
OLMALINC	Oligodendrocyte maturation-associated long intergenic non-coding RNA	5.74	6.04E-149
ABLIM2	Actin binding LIM protein family member 2	5.70	1.61E-88
GPR153	G protein-coupled receptor 153	5.57	9.86E-124
ACSM3	Acyl-CoA synthetase medium chain family member 3	5.56	3.35E-37
DGAT2	Diacylglycerol O-acyltransferase 2	5.47	5.00E-127
NCAM2	Neural cell adhesion molecule 2	5.40	1.62E-34
ECHDC1	Ethylmalonyl-CoA decarboxylase 1	5.31	2.84E-213
FLVCR1-DT	FLVCR1 divergent transcript	5.29	5.45E-11
IFI27	Interferon alpha inducible protein 27	5.23	1.58E-14
FASN	Fatty acid synthase	5.18	4.50E-133
EPHB2	EPH receptor B2	5.18	7.43E-79
EPB41L4A	Erythrocyte membrane protein band 4.1 like 4A	5.13	4.54E-12

PLEKHH1	Pleckstrin homology, MyTH4 and FERM domain containing H1	5.05	1.40E-129
ELOVL5	ELOVL fatty acid elongase 5	5.04	1.14E-203
PLCD4	Phospholipase C delta 4	4.99	7.48E-23
CKMT2	Creatine kinase, mitochondrial 2	4.95	1.27E-136
STX11	Syntaxin 11	4.86	2.08E-134
PCBD1	Pterin-4 alpha-carbinolamine dehydratase 1	4.78	2.55E-141
TSPAN13	Tetraspanin 13	4.76	8.38E-150
SLC2A6	Solute carrier family 2 member 6	4.71	7.23E-99
HMGCS1	3-hydroxy-3-methylglutaryl-CoA synthase 1	4.65	1.25E-55
CABLES1	Cdk5 and Abl enzyme substrate 1	4.65	7.29E-195
RPH3AL	Rabphilin 3A like (without C2 domains)	4.59	1.56E-22
CD9	CD9 molecule	4.57	4.93E-133
LINC01128	Long intergenic non-protein coding RNA 1128	4.52	1.61E-37
LETM1	Leucine zipper and EF-hand containing transmembrane protein 1	4.52	4.68E-205
C1orf54	Chromosome 1 open reading frame 54	4.42	1.88E-30
ADIPOR2	Adiponectin receptor 2	4.34	1.36E-237
ADIRF	Adipogenesis regulatory factor	4.30	1.48E-83
UBASH3B	Ubiquitin associated and SH3 domain containing B	4.30	4.10E-143
RIPOR2	RHO family interacting cell polarization regulator 2	4.27	2.48E-09
ACSL4	Acyl-CoA synthetase long chain family member 4	4.13	1.02E-66
FAM83D	Family with sequence similarity 83 member D	4.06	2.15E-04
ANK3	Ankyrin 3	4.05	1.86E-33
CLSTN3	Calsyntenin 3	4.00	6.73E-60
DENND3	DENN domain containing 3	4.00	2.28E-120
ZFAT	Zinc finger and AT-hook domain containing	4.00	3.34E-160
RASSF2	Ras association domain family member 2	4.00	2.95E-55
MECR	Mitochondrial trans-2-enoyl-CoA reductase	3.99	1.60E-104
AGPAT2	1-acylglycerol-3-phosphate O-acyltransferase 2	3.99	2.42E-107
DHCR7	7-dehydrocholesterol reductase	3.98	6.07E-114
KIAA1671	KIAA1671	3.93	3.84E-46
ABCA4	ATP binding cassette subfamily A member 4	3.86	6.26E-12
SLC19A1	Solute carrier family 19 member 1	3.86	2.33E-87
BRINP2	BMP/retinoic acid inducible neural specific 2	3.82	1.26E-25
LDB3	LIM domain binding 3	3.74	1.85E-19
PRKCD	Protein kinase C delta	3.70	2.13E-126
NKILA	NF-kappaB interacting lncRNA	3.69	1.41E-08
OSBPL11	Oxysterol binding protein like 11	3.63	5.82E-113
LRRC2	Leucine rich repeat containing 2	3.57	4.69E-08
SPIRE2	Spire type actin nucleation factor 2	3.56	9.59E-20
SULT4A1	Sulfotransferase family 4A member 1	3.51	6.48E-08
GCH1	GTP cyclohydrolase 1	3.48	1.05E-21

KCNQ3	Potassium voltage-gated channel subfamily Q member 3	3.43	3.31E-63
DIAPH3	Diaphanous related formin 3	3.42	7.71E-09
CDK15	Cyclin dependent kinase 15	3.36	5.65E-68
FLVCR2	Feline leukemia virus subgroup C cellular receptor family member 2	3.23	3.88E-57
MAPK8IP2	Mitogen-activated protein kinase 8 interacting protein 2	3.19	6.93E-34
DMTN	Dematin actin binding protein	3.14	3.95E-48
EGR3	Early growth response 3	2.95	4.32E-09
EXOSC10-AS1	EXOSC10 antisense RNA 1	2.86	5.81E-03
PRPS2	Phosphoribosyl pyrophosphate synthetase 2	2.78	1.55E-55
CLDN7	Claudin 7	2.70	2.30E-02
NETO2	Neuropilin and tolloid like 2	2.64	3.03E-04
GRK3	G protein-coupled receptor kinase 3	2.61	2.15E-43
CMYA5	Cardiomyopathy associated 5	2.56	1.86E-38
MAP6	Microtubule associated protein 6	2.48	1.74E-02
GKAP1	G kinase anchoring protein 1	2.47	4.59E-08
AKAP1	A-kinase anchoring protein 1	2.46	1.01E-75
BLM	BLM RecQ like helicase	2.39	3.27E-10
TAF1A-AS1	TAF1A antisense RNA 1	2.39	1.19E-06
BRIP1	BRCA1 interacting protein C-terminal helicase 1	2.31	3.01E-03
AGAP11	ArfGAP with GTPase domain, ankyrin repeat and PH domain 11	2.16	7.38E-13
IFI30	IFI30 lysosomal thiol reductase	2.01	1.19E-05

Table S2. Top 15 GO terms for 137 FADAGs.

Category	Term name	P-value	Benjamini
GOTERM_BP_FAT	Lipid metabolic process	1.10E-16	2.90E-13
GOTERM_BP_FAT	Cellular lipid metabolic process	3.30E-15	4.50E-12
GOTERM_BP_FAT	Lipid biosynthetic process	2.30E-14	2.10E-11
GOTERM_BP_FAT	Thioester metabolic process	3.40E-12	1.90E-09
GOTERM_BP_FAT	Acyl-CoA metabolic process	3.40E-12	1.90E-09
GOTERM_BP_FAT	Fatty acid metabolic process	1.10E-11	5.10E-09
GOTERM_BP_FAT	Monocarboxylic acid metabolic process	5.20E-11	2.00E-08
GOTERM_BP_FAT	Coenzyme metabolic process	8.30E-11	2.80E-08
GOTERM_BP_FAT	Triglyceride metabolic process	1.60E-10	4.90E-08
GOTERM_BP_FAT	Small molecule biosynthetic process	2.10E-10	5.80E-08
GOTERM_BP_FAT	Coenzyme biosynthetic process	4.00E-10	9.80E-08
GOTERM_BP_FAT	Fatty acid biosynthetic process	4.70E-10	1.10E-07
GOTERM_BP_FAT	Monocarboxylic acid biosynthetic process	6.90E-10	1.20E-07
GOTERM_BP_FAT	Acylglycerol metabolic process	7.10E-10	1.20E-07
GOTERM_BP_FAT	Acyl-CoA biosynthetic process	7.60E-10	1.20E-07

Table S3. Information on human dermal fibroblasts (HDFs) and mesenchymal stem cells (MSCs) used in this study.

Abbreviation	Lot#	Passage	BMI	Age	Gender	Ethnicity	Site
HDF38	DFM090214A	2	23.1	38	Male	Caucasian	Abdomen
HDF35	DFM102715B	3	33.9	35	Female	Hispanic	Abdomen
HDF37	DFM111714A	3	33.1	37	Female	African-American	Abdomen
HDF44	DFM101014C	2	31.8	44	Female	African-American	Breast
HDF54	DDFM052010B	3	21.3	54	Female	African-American	Abdomen
AdMSC46	394Z027.1	2	Unknown	46	Female	Caucasian	Abdomen
AdMSC51	423Z037.1	2	24	51	Female	Caucasian	Breast
BmMSC70	429A022	2	Unknown	70	Male	Caucasian	Femoral head

Table S4. Sequences and product size of primers used for qRT-PCR.

Gene	Sense primer	Antisense primer	Product size
TBP	CCTAAAGACCATTGCACTTCGTG	GTGGTTCGTGGCTCTCTTATC	93
UCP1	TCTACGACACGGTCCAGGAG	GAATACTGCCACTCCTCCAGTC	104
FABP4	GCCAGGAATTTGACGAAGTCA	CCCATTTCTGCACATGTACCAG	93
CIDEA	AAGGCCACCATGTATGAGATGTAC	ACAGGAACCGCAGCAGACTC	91
GPAM	GATCGCATTATCGAAGGTCACTAC	TTGCTACACTCCACAGGCTCTC	79
LIPE	CTCAGTGTGCTCTCCAAGTG	CTTTCTGGTCTGAGTTGGAGTG	76
CKMT1	AGCAGGAATGGCTCGAGAC	ATCCTCCTCATTCACCCAGATC	88
ADIPOQ	CTGGTGAGAAGGGTGAGAAAG	GTTTCACCGATGTCTCCCTTAG	64
CD36	CAACCTATTGGTCAAGCCATCAG	TGTTTGCCTTCTCATCACCAATG	119
SREBF1	CCTGGTCTACCATAAGCTGCAC	CTGCACACTCTGCCAGGTTC	110
FASN	CCTTCGAGGTGTCAGAGAAC	TTTCCGGGTGGTCGAAGAG	93
GPD1	CTGACCTGATCACTACCTGCTATG	GCAACTCTTTCTCCAGCTGCTC	99
GK	GATTTGTCTGCCGTCACGATG	CCAACCCATTGACTTCATCACAG	108
KCNK3	CCATCACCGTCATCACCAC	CAGCGCGTAGAACATGCAG	86
ADRB2	CTGCTGTGACTTCTTCACGAAC	AGACCCTGGAGTAGACGAAGAC	101
AEBP1	AACGCCACCATCTCTGTGAG	CAAGATTCGCCAGTAATCACCAC	75
CEBPA	AACATCGCGGTGCGCAAGA	AGCTCCAGCACCTTCTGCT	74
PPARGC1A	AGGTCAAGATCAAGGTCTCCAG	GGTGTCTGTAGTGGCTTGACTC	79
PPARGC1B	AGTGTTTGGTGAGATTGAGGAGTG	AGAACACCGGTAGGTGATGAAG	85
PPARG	TGGAATTAGATGACAGCGACTTG	CTTCAATGGGCTTCACATTCAG	93
RXRG	GGCCTACACCAAGCAGAAGTATC	CAAGCCAATGGAACGCAGAG	88
LXRA	CAGATTGCCCTGCTGAAGAC	AGGGTTGTACCTCCGAGATG	69
CITED1	TGGCACCTCACCTGCGAAG	GCAGAATGGCCACTGCTTTG	98
